# Matrix Metalloproteinase‐8 Deficiency Alters the Balance Between Viral Clearance and Immunopathology in H1N1 Infection

**DOI:** 10.1096/fj.202601719RRR

**Published:** 2026-10-12

**Authors:** Xiaoyun Wang, Joselyn Rojas‐Quintero, Duo Zhang, Joaquin Zuñiga, Caroline A. Owen

**Affiliations:** ^1^ Pulmonary Division, Department of Medicine Baylor College of Medicine Houston Texas USA; ^2^ Division of Pulmonary and Critical Care Medicine Brigham and Women's Hospital, and Harvard Medical School Boston Massachusetts USA; ^3^ Clinical and Experimental Therapeutics, College of Pharmacy University of Georgia and Charlie Norwood VA Medical Center Augusta Georgia USA; ^4^ Department of Immunology Instituto Nacional de Enfermedades Respiratorias Ismael Cosío Villegas Mexico City Mexico; ^5^ Tecnologico de Monterrey, Escuela de Medicina y Ciencias de la Salud Mexico City Mexico

**Keywords:** adaptive immunity, innate immunity, murine models of influenza viral infection, necroptosis, Type I interferons

## Abstract

Matrix metalloproteinase‐8 (MMP‐8) regulates inflammatory responses, but its contributions to the pathogenesis of influenza A viral (IAV) disease are not known. Plasma MMP‐8 levels were increased 30‐fold in subjects infected with H1N1 or seasonal IAV and were inversely associated with the PaO_2_/FiO_2_ ratio, suggesting potential value as a candidate marker of lung injury severity that requires validation in larger cohorts. MMP‐8 levels were elevated in the lungs of H1N1‐infected wild‐type (WT) mice and localized to airway epithelial cells, macrophages, and polymorphonuclear neutrophils (PMNs). H1N1‐infected *Mmp‐8* ^*−/−*^ mice exhibited lower mortality and reduced lung viral burdens, but developed more severe lung injury than WT mice. Gene expression profiling revealed enhanced necroptosis and an exaggerated immune response in the lungs of infected *Mmp‐8* ^*−/−*^ mice. Infected *Mmp‐8* ^*−/−*^ mice had increased airway epithelial necroptosis with higher pulmonary levels of Type I interferons (IFNs), and increased frequencies of Class II MHC molecule‐expressing macrophages and CD4^+^ and CD8^+^ T cells, and higher levels of some Th1 cytokines. In vitro, MMP‐8 cleaved IL‐1β and IL‐6, but not Type I IFNs. These findings suggest that *Mmp‐8* deficiency is associated with restricted influenza virus spread and increased lung injury, accompanied by enhanced Type I IFN‐associated necroptotic signaling and augmented antiviral immune responses. The latter may partly involve loss of MMP‐8‐mediated processing of pro‐inflammatory mediators. MMP‐8 may represent a host‐response target in severe influenza infection, although therapeutic strategies targeting MMP‐8 will need to balance enhanced viral clearance against the risk of increased lung immunopathology.

## Graphical abstract

Mmp‐8 deficiency during H1N1 influenza infection enhances Type I interferon responses, necroptosis‐associated signaling, and adaptive immunity, leading to lower lung viral burden and improved survival, but also amplifies inflammatory lung injury.

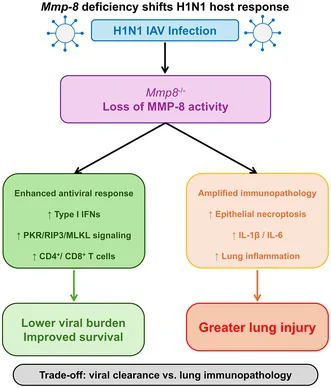

**Abbreviations**
ALI: acute lung injury
BAL: bronchoalveolar lavage
BALF: BAL fluid
DAMPs: damage‐associated molecular patterns
Eif2ak2: eukaryotic translation initiation factor 2 alpha kinase 2
IAV: influenza A viral
IL‐1β: interleukin‐1β
IL‐6: interleukin‐6
LPS: lipopolysaccharide
MMP‐8: matrix metalloproteinase‐8
MMP‐9: matrix metalloproteinase‐9
MPO: myeloperoxidase
Pan‐CK: pan‐cytokeratin
PFU: plaque‐forming units
PRRs: pattern recognition receptors
rhMMP‐8: recombinant human MMP‐8
WT: wild‐type

## 1 Introduction

Influenza A viral (IAV) infections are associated with high morbidity and mortality [1]. Complications associated with IAV, including secondary bacterial pneumonia and cytokine storm, can lead to multi‐organ failure and increased mortality [1]. Although vaccines are developed annually for emerging IAV strains, not all subjects are vaccinated or mount robust protective immune responses to the vaccines [2]. Antiviral drugs (such as oseltamivir, which inhibits viral neuraminidase) are not fully effective in treating IAV infections or reducing IAV‐associated mortality, especially when there is a delay in initiating therapy [3, 4]. Thus, there is an urgent need to identify new targets to develop more effective therapies for IAV infections, including therapies directed against host factors that promote the development of more severe disease.

Matrix metalloproteinase‐8 (MMP‐8) belongs to the structurally related family of Zn^2+^‐dependent MMPs, which regulate inflammation [5, 6, 7]. MMP‐8 is most highly expressed by polymorphonuclear neutrophils (PMNs) [5, 8]. Pro‐MMP‐8 is produced in PMN precursors in the bone marrow and stored in and subsequently released from the secondary granules of PMNs [5]. Pro‐MMP‐8 is released by PMNs into the extracellular space and then activated by the cysteine switch mechanism. MMP‐8 is also produced in smaller amounts by activated macrophages, vascular smooth muscle cells, and bronchial epithelial cells [9, 10, 11].

MMP‐8 promotes pulmonary fibrosis in mice [7, 8, 9]. MMP‐8 has pro‐inflammatory activities during the systemic inflammatory response syndrome [12], in cardiovascular disease [13], and neuroinflammation [14]. MMP‐8 also promotes inflammation in other organs in experimental animal models [15, 16]. However, *Mmp‐8* deficiency in mice leads to greater pulmonary inflammation and injury during lipopolysaccharide (LPS) and bleomycin‐mediated lung inflammation due to loss of *Mmp‐8*‐mediated proteolytic inactivation of Mip‐1α [6, 7] and allergen‐induced airway inflammation via loss of *Mmp‐8*‐induced granulocyte apoptosis [17].

Little is known about the contributions of MMP‐8 to IAV infection. Recently, *Mmp‐8* expression was reported to be highly induced in whole lung samples throughout IAV (A/Puerto Rico/8/1934 [H1N1]) infection using genome‐wide RNA sequencing [18]. Thus, we tested the hypotheses that: (1) MMP‐8 levels are increased in plasma and/or lung samples from IAV‐infected human subjects and/or wild‐type (WT) mice; and (2) *Mmp‐8* deficiency alters host responses to IAV infection, thereby affecting viral clearance and lung injury.

## 2 Materials and Methods

### 2.1 Materials

ELISA kits to measure human MMP‐8, murine interleukin (IL)‐6, CCL5, IL‐10, IL‐1β, CCL‐2, tumor necrosis factor‐α (TNF‐α), IL‐17, and transforming growth factor‐β (TGF‐β) were obtained from R&D Systems (Minneapolis, MN). ELISA kits to quantify murine granulocyte colony‐stimulating factor (G‐CSF), granulocyte macrophage CSF (GM‐CSF), macrophage colony‐stimulating factor (M‐CSF), macrophage inflammatory protein‐1α (MIP‐1α; CCL‐3), MIP‐2 (CXCL‐2), and keratinocyte‐derived chemokine (KC; CXCL‐1) were purchased from Peprotech (Rocky Hill, NJ). ELISA kits to measure murine granulocyte chemotactic protein‐2 (GCP‐2; CXCL‐6) and MMP‐8 were purchased from MyBioSource (San Diego, CA). ELISA kits to measure murine interferon (IFN)‐α and IFN‐γ were purchased from Affymetrix eBioscience (San Diego, CA). ELISA kits to quantify murine IFN‐β were purchased from PBL Assay Science (Piscataway, NJ). Recombinant human MMP‐8 was purchased from R & D Systems (Minneapolis, MN). GM6001 was purchased from EMD Millipore (Billerica, MA). 1,10‐phenanthroline (OP) was purchased from Sigma‐Aldrich (St. Louis, MO). Recombinant human IFN‐β, CCL‐2, CCL‐5, IL‐8, IL‐6, and IL‐1β were purchased from Peprotech (Rocky Hill, NJ). Recombinant human IFN‐α2α and IFN‐α2β were purchased from STEMCELL Technologies (Cambridge, MA).

### 2.2 Human Subjects

All studies with human subjects were approved by the Institutional Review Boards (IRB) of the Instituto Nacional de Ciencias Médicas y Nutrición Salvador Zubirán (INCMNSZ, Approval Number: 3349) and the Instituto Nacional de Enfermedades Respiratorias Ismael Cosío Villegas (INER, Approval Number: B28‐16 and B09‐20), Mexico City, and Brigham and Women's Hospital (2002‐P‐000253; BWH), Boston. Informed consent was obtained from all participants [19, 20]. Table 1 shows the demographic and clinical data on these subjects.

**TABLE 1:** Demographic and clinical characteristics of the human cohort.

Characteristics	Uninfected healthy volunteers (*N* = 17)	Seasonal IAV infection (*N* = 19)	H1N1 IAV infection (*N* = 57)	*p*
Males (%)^a^	11 (64)	8 (42)	33 (66)	NS
Age (years)^a^	41 ± 8	39 ± 11	43 ± 13	NS
Influenza vaccination, *n* (%)^a^	14 (82)	0 (0)	3 (5)	0.001^b^
Diagnosis of diabetes mellitus, *n* (%)^a^	0 (0)	1 (5)	8 (14)	NS
Obese, *n* (%)^a^, ^c^	1 (6)	7 (37)	24 (42)	0.02^d^
PaO_2_:FiO_2_ ^e^	N/A	211 (101–284)	157 (50–309)	0.01^e^

*Note:* Plasma samples from patients infected with seasonal IAV or H1N1 IAV were provided by Dr. Joaquin Zuñiga from the Instituto Nacional de Enfermedades Respiratorias Ismael Cosío Villegas, Mexico City. The samples from patients infected with seasonal influenza were taken within the first 2 weeks of the onset of symptoms. For subjects infected with H1N1, the samples were collected during the first 30 days after they were admitted to the intensive care unit. Data are presented as median (interquartile range) for non‐parametric data or mean ± SD for parametric data.

Abbreviations: IAV, influenza A virus; NS, not significant.

^a^ Categorical variables were analyzed with the Z‐proportions test. Non‐categorical data were analyzed using one‐way ANOVAs followed by Brown‐Forsythe equal variance test. Pairwise comparisons for parametric data were performed using Student's *t*‐tests. NS, not significantly different.

^b^ The proportion of uninfected controls that had been vaccinated against IAV within 12 months from obtaining the plasma samples was greater than that of subjects infected with seasonal IAV and H1N1 (*p* < 0.001 for both comparisons).

^c^ Obesity was defined as body mass index > 30 kg/m^2^.

^d^ The proportion of obese subjects infected with seasonal IAV and H1N1 IAV was greater than that of uninfected obese subjects (*p* = 0.029 and *p* = 0.006, respectively).

^e^ PaO_2_:FiO_2_ ratio is a marker of the severity of acute lung injury/adult respiratory distress syndrome. PaO_2_:FiO_2_ ratio of subjects infected with H1N1 was significantly lower than that in subjects infected with seasonal IAV (*p* = 0.01).

### 2.3 Plasma MMP‐8 Levels

Plasma samples obtained from patients diagnosed with seasonal IAV and pandemic H1N1 were provided by Dr. Joaquin Zuñiga. These study subjects have been described in detail previously [20]. Human plasma MMP‐8 levels were measured in duplicate using a commercially available ELISA kit (R & D Systems, Minneapolis, MN).

### 2.4 Animal Studies

All studies conducted on mice were approved by the Institutional Care and Use Committee at Brigham and Women's Hospital (Protocol No. 3501) and the Charlie Norwood Veterans Affairs Medical Center Institutional Animal Care and Use Committee (Protocol No. 24‐08‐128). The authors followed ARRIVE (Animal Research: Reporting of In Vivo Experiments) guidelines for reporting on animal studies.

### 2.5 Mice

Mice were housed in a barrier facility under specific pathogen‐free conditions. *Mmp‐8* ^*−/−*^ mice in a pure C57BL/6 background were studied [6, 7]. The *Mmp‐8* ^*−/−*^ mice have a normal lifespan, fertility, and lung development, and no abnormality has been identified in any organ in the unchallenged state [16]. C57BL/6 WT parental littermate mice were used as controls for *Mmp‐8* ^*−/−*^ mice.

### 2.6 Cell Lines and Virus Stocks

Madin‐Darby canine kidney (MDCK, CCL‐34) cells (American Tissue Culture Collection, Manassas, VA) were maintained in minimum essential medium (MEM) supplemented with L‐glutamine, Earle's salts, 10% fetal bovine serum, 100 U/mL penicillin, 100 μg/mL streptomycin, and 0.25 μg/mL amphotericin B (ThermoFisher Scientific, Waltham, MA). The pandemic IAV strain, influenza A/California/07/2009 H1N1 (H1N1), was obtained from the Centers for Disease Control (CDC, Atlanta, GA), propagated in Madin‐Darby Canine Kidney (MDCK) cells, and used to infect mice. Titers of stock were measured using a standard plaque assay on 6‐well plates of confluent monolayers of MDCK cells as described previously [19, 21] using a 0.6% Avicel CL‐611 solution (FMC Corporation, Philadelphia, PA) containing 3 μg/mL of trypsin solution treated with 1‐tosylamido‐2‐phenylethyl chloromethyl ketone (Worthington Biochemical Corporation, Lakewood, NJ).

### 2.7 IAV Infection of Mice

Adult C57BL/6 strain WT and *Mmp‐8* ^*−/−*^ mice were infected with A/California/04/2009 H1N1 virus through intranasal instillation [19]. An LD_80_ inoculum (10^4^ plaque‐forming units [PFU]), resulting in 80% mortality in WT mice, was used to evaluate survival and loss of body weight. A lower LD_20_ inoculum (10^3^ PFU) was used for experiments requiring tissue harvest, including measurements of lung viral titers, lung inflammation, acute lung injury (ALI), respiratory mechanics, cytokine levels, and immunostaining endpoints to ensure reproducible infection while preserving sufficient numbers of surviving mice for analysis at the indicated time points. The LD_80_ and LD_20_ inocula were selected based on preliminary dose–response studies and our previously established infection model [19].

### 2.8 *Mmp‐8* Levels

*Mmp‐8* levels were measured using quantitative real‐time PCR or a commercial ELISA kit [22]. Lung sections were double immunostained for MMP‐8 and markers of alveolar macrophages (AMs; MAC‐3), lung epithelial cells (pancytokeratin; Pan‐CK), or PMNs (myeloperoxidase; MPO).

### 2.9 Leukocyte and Lymphocyte Subset Enumeration in Bronchoalveolar Lavage (BAL) Samples

WT and *Mmp‐8* ^*−/−*^ mice were infected with a 10^3^ PFU H1N1 by the intranasal route and were euthanized at Days 3, 7, and 10 post‐infection (p.i.). Uninfected mice were used as controls. BAL was performed on other cohorts of mice using 8 × 0.5 mL aliquots of PBS [19]. BAL leukocytes were then stained for surface antigens for 30 min at 4°C in PBS containing 2% fetal calf serum and fixed using the Fixation/Permeabilization buffer set (BD Biosciences) following the manufacturer's instructions. Antibodies were purchased from Biolegend (San Diego, CA), eBioscience (San Diego, CA), and BD Biosciences (San Jose, CA). Flow cytometry was performed on a BD LSR Fortessa Cell Analyzer (San Jose, CA) and analyzed with FlowJo software (Tree Star Inc.).

### 2.10 Quantification of Mediators of Inflammation and IFNs in Lung Samples

Murine lungs were homogenized in 1 mL of PBS containing 0.5% Triton X‐100 and 1 mM phenylmethylsulfonyl fluoride, 1 mM 1,10‐phenanthroline, and Mammalian Protease Inhibitor Cocktail (Sigma‐Aldrich, St. Louis, MO). Commercially available ELISA kits were used to measure IL‐1β, IL‐6, IL‐10, CCL‐2, CCL‐5, IL‐17, TNF‐α AND TGF‐β (R & D SYSTEMS, MINNEAPOLIS, MN), G‐CSF, GM‐CSF, M‐CSF, MIP‐1α, MIP‐2, KC (CXCL‐1) (Peprotech, Rocky Hill, NJ), GCP‐2 (CXCL‐6) (MyBioSource, San Diego, CA), IFN‐α and IFN‐γ (Affymetrix/eBioscience, San Diego, CA) and IFN‐β (PBL Assay Science, Piscataway, NJ). Levels of each analyte were normalized to the total lung protein level measured using a commercial kit (Thermo Fisher Scientific, Waltham, MA).

### 2.11 Lung Viral Titers

Viral RNA copy number was measured using quantitative real‐time reverse‐transcription PCR [19]. RNA was extracted from H1N1‐infected lung tissue using a SurePrep TrueTotal RNA Purification Kit (Fisher Scientific, Fair Lawn, NJ). RNA (1 μg) was reverse transcribed into cDNA using a High‐Capacity cDNA Reverse Transcription Kit (ThermoFisher Scientific, Carlsbad, CA). Quantitative PCR was performed to amplify H1N1 cDNA using an AriaMx Real‐Time PCR machine (Agilent Technologies, Santa Clara, CA) and primers (ThermoFisher Scientific, Waltham, MA) for H1N1 IAV (Primer sequences are shown in Table S1). Viral copy numbers were calculated using H1N1 DNA standards of known concentrations and expressed as H1N1 viral RNA copies per μg of total lung RNA.

### 2.12 ALI

To measure the permeability of the alveolar‐capillary barrier in the murine lung, mice were infected with 10^3^ PFU H1N1 by the intranasal route and euthanized on Days 3, 7, and 10 p.i. Uninfected mice were used as controls. BAL was performed with one aliquot of 0.5 mL of PBS, and albumin was measured in BAL fluid (BALF) samples using an ELISA (GenWay Biotech Inc., San Diego, CA) [23].

### 2.13 Respiratory Mechanics

To evaluate lung injury, mice were infected with 10^3^ PFU H1N1 by the intranasal route for 7 days p.i. Uninfected mice were used as controls. Mice were anesthetized using a cocktail of 100 mg/kg ketamine, 10 mg/kg xylazine, and 3 mg/kg acepromazine delivered by the intraperitoneal route. A tracheostomy was performed, and an 18‐gauge cannula was inserted and secured in the trachea using sutures. The animals were connected by the tracheal cannula to a digitally controlled mechanical ventilator (a FlexiVent device; Scireq Inc., Montreal, Quebec, Canada). Ventilator settings were f = 150/min, FiO_2_ = 0.21, tidal volume = 10 mL/kg body weight, and a positive end‐expiratory pressure (PEEP) of 3 cm of H_2_O. Murine lungs were inflated to total lung capacity (25 cm H_2_O) three times, and then tissue elastance and peripheral airway elastance were measured at a PEEP of 3 cm H_2_O, followed by stepwise quasi‐static compliance (Cst), and volume‐pressure flow curves [24]. After the measurements of respiratory mechanics were completed, mice were humanely euthanized.

### 2.14 Necrostatin‐1 (RIP1 Inhibitor) Treatment in H1N1‐Infected *Mmp‐8* ^*−/−*^ Mice

*Mmp‐8* ^*−/−*^ mice were infected intranasally with 10^3^ PFU H1N1 influenza virus. At 24 h after infection, mice received an intraperitoneal injection of Necrostatin‐1 (Nec‐1; catalog #480065, Sigma‐Aldrich) at a dose of 6 mg/kg body weight [25, 26]. Necrostatin‐1 was prepared as a 60 mg/mL stock solution in DMSO and diluted 1:100 in saline to a working concentration of 0.6 mg/mL, corresponding to a final DMSO concentration of 1%. Control mice received an equal volume of vehicle containing 1% DMSO in saline. Mice were euthanized on Day 3 p.i. BAL fluid albumin concentrations were measured using an ELISA kit (GenWay Biotech Inc., San Diego, CA). Lung viral titers were quantified by quantitative real‐time reverse‐transcription PCR. H1N1 viral copy numbers were calculated using H1N1 DNA standards of known concentrations.

### 2.15 Mouse Toll‐Like Receptor (TLR) Signaling Pathway PCR Array

WT and *Mmp‐8* ^*−/−*^ mice were infected with 10^3^ PFU H1N1 by the intranasal route and euthanized at Days 1, 3 and 7 p.i. Total RNA was isolated from lung samples using a SurePrep TrueTotal RNA Purification Kit (Fisher Scientific, Fair Lawn, NJ), following the manufacturer's instructions. For each experimental group and time point, equal amounts of lung RNA from 6 to 8 mice were pooled before PCR array analysis. 1 μg of pooled RNA was reverse transcribed into cDNA using a High‐Capacity cDNA Reverse Transcription Kit (Thermo Fisher Scientific, Carlsbad, CA). RT^2^ Profiler mouse TLR signaling pathway PCR Array (QIAGEN, Germany) was used to measure the expression of 84 genes central to TLR‐mediated signal transduction and innate immunity. A heat map was generated using hierarchical cluster analysis to show TLR‐mediated signal transduction and innate immunity gene expression patterns in lung tissues between WT and *Mmp‐8* ^*−/−*^ mice after IAV infection. Because each group and time point was represented by a single pooled RNA sample without independent biological replicates, the array data were analyzed descriptively without inferential statistical testing. A greater than three‐fold change was used as an exploratory screening threshold to identify genes showing the largest expression differences for subsequent evaluation.

### 2.16 RT‐PCR Measurements of Eif2ak2 (Pkr), Ifn‐α2, Ifn‐α4, Ifn‐β, Ifn‐γ, Receptor‐Interacting Serine/Threonine‐Protein Kinase 1 (Rip1) and Rip3

WT and *Mmp‐8* ^*−/−*^ mice were infected with 10^3^ PFU H1N1 by the intranasal route and euthanized at Days 1, 3 and 7 p.i. Uninfected mice were used as controls. Total RNA was isolated from lung samples using a SurePrep TrueTotal RNA Purification Kit (Fisher Scientific, Fair Lawn, NJ), following the manufacturer's instructions, and 1 μg of RNA was reverse transcribed into cDNA using a High‐Capacity cDNA Reverse Transcription Kit (Thermo Fisher Scientific, Carlsbad, CA). SYBR green‐based real‐time RT‐PCR was used to measure *Eif2ak2* (*Pkr*), *Ifn‐α2*, *Ifn‐α4*, *Ifn‐β*, *Ifn‐γ*, *Rip1* and *Rip3* gene expression using primers from Invitrogen (Charlestown, MA), *Ppia* and/or *β‐actin* as the housekeeping genes (see Table S1 for primer sequences), the comparative threshold method, and an AriaMx Real‐time PCR machine (Agilent Technologies, Santa Clara, CA) [22].

### 2.17 Double Immunofluorescence Staining of Murine Lung Sections for Readouts of Necroptosis (Phospho‐RIP3 [p‐RIP3] and Phospho‐Mixed Lineage Kinase Domain Like Pseudokinase [p‐MLKL])

Formalin‐fixed lung sections from mice were double immunostained for p‐RIP3 and markers of airway epithelial cells (pancytokeratin; Pan‐CK). Briefly, lung sections were immunostained with rabbit anti‐murine p‐RIP3‐Ser^232^ IgG (Abcam, Cambridge, MA) followed by goat anti‐rabbit F(ab′)_2_ conjugated to Alexa‐488. Lung sections were also immunostained with the following mouse anti‐murine pancytokeratin IgG2a (Sigma‐Aldrich, St. Louis, MO) followed by goat anti‐murine F(ab′)2 conjugated to Alexa‐546. All secondary antibodies were obtained from Invitrogen (Carlsbad, CA). Isotype‐matched non‐immune rabbit and murine IgGs were used as control primary antibodies. Lung sections were counterstained with 4′,6‐diamidino‐2‐phenylindole (DAPI). Images of the immuno‐stained lung sections were captured and analyzed using a confocal microscope (Olympus Corporation, Center Valley, PA). For each lung section, immunostaining in epithelial cells was quantified in 10 randomly acquired images using MetaMorph software (Molecular Devices, Sunnyvale, CA), and the staining signal was normalized to the unit area of bronchial wall evaluated.

### 2.18 Quantification of MLKL and RIP3 Activation (Phosphorylation) in Lung Samples

WT and *Mmp‐8* ^*−/−*^ mice were infected with 10^3^ PFU H1N1 by the intranasal route and were euthanized on Day 3 p.i. Lungs were removed and homogenized in RIPA buffer containing 1 mM phenylmethylsulfonylfluoride, 1 mM 1,10‐phenanthroline, 1% Sigma Mammalian Protease Inhibitor Cocktail and PhosSTOP (Sigma, St. Louis, MO). The total protein concentration was determined using a BCA protein assay kit (Thermo‐Fisher Scientific, Rockford, IL). Protein lysates were separated on 12% SDS‐PAGE gels, transferred to PVDF membranes, and then incubated with rabbit anti‐MLKL IgG (Abcam, Cambridge, MA), rabbit anti‐Ser^345^ phospho‐MLKL IgG (Abcam, Cambridge, MA), rabbit anti‐RIP3 IgG (Abcam, Cambridge, MA), rabbit anti‐p‐RIP3‐Ser^232^ IgG (Abcam, Cambridge, MA), or mouse anti‐α‐Tubulin IgG (Sigma‐Aldrich, St. Louis, MO). Membranes were then incubated with goat anti‐rabbit IgG (H + L) conjugated to HRP or goat anti‐murine IgG (H + L) conjugated to HRP (Bio‐Rad, Hercules, CA), and signals were developed using a chemiluminescence kit. Western blot images were captured using a ChemiDoc imaging system and analyzed by densitometry (Bio‐Rad, Hercules, CA). Phosphorylated MLKL (p‐MLKL‐Ser^345^) and phosphorylated RIP3 (p‐RIP3‐Ser^232^) levels were normalized to total MLKL or RIP3 levels, respectively.

### 2.19 Cleavage Assays

Human recombinant MMP‐8 (rhMMP‐8) was activated as described previously [8, 27]. Human IFN‐α2 (1000 nM), IFN‐β (1000 nM), IL‐6 (900 nM), CCL‐2 (2000 nM), CCL‐5 (2000 nM), IL‐8 (2750 nM), or IL‐1β (1100 nM) were incubated in a total volume of 20 μL of assay buffer (50 mM Tris, 10 mM CaCl_2_, 150 mM NaCl, 0.05% (w/v) Brij35, pH 7.5) with or without 0.3 μM active rhMMP‐8 either with or without 1 mM 1,10‐phenanthroline (a non‐selective inhibitor of metalloproteinases that efficiently inhibits MMP‐8) for 18 h at 37°C. Reaction products were separated on Tris‐tricine gels and visualized using a silver staining kit (Thermo Fisher Scientific, Fair Lawn, NJ).

### 2.20 Primary Airway Epithelial Cell Isolation and Recombinant Human IFN‐β (rhIFN‐β) Treatment

Primary airway epithelial cells were isolated from WT and *Mmp‐8* ^*−/−*^ mice as previously described [28]. Primary cells were cultured in Airway Epithelial Cell Basal Medium (ATCC, Manassas, VA). Upon reaching the appropriate confluence, primary airway epithelial cells were treated with 20 ng/mL rhIFN‐β (Peprotech, Rocky Hill, NJ) for 24 h. Following treatment, *Eif2ak2* (*Pkr*) mRNA expression was quantified by RT‐qPCR. To evaluate necroptotic signaling, immunofluorescence staining was performed to detect phosphorylated RIP3 (p‐RIP3) and phosphorylated MLKL (p‐MLKL).

### 2.21 Statistics

SigmaPlot (Systat Software, San Jose, CA) was used for statistical analyses. Data that are normally distributed are presented as mean ± SD or SEM and analyzed using one‐way ANOVA tests followed by pair‐wise comparisons using two‐tailed Student's *t*‐tests. Non‐normally distributed data are presented as box plots, with boxes showing the medians and 25th and 75th percentiles and whiskers showing the 10th and 90th percentiles. These data were analyzed using one‐way ANOVA followed by pairwise Mann–Whitney *U* tests. *p* ≤ 0.05 was considered to be statistically significant. To assess the association between plasma MMP‐8 levels and the PaO_2_/FiO_2_ ratio, multivariable linear regression analyses were conducted exclusively among influenza‐infected subjects. Healthy controls were not included because PaO_2_/FiO_2_ measurements were not available for this group. The adjusted analyses were conducted using complete cases, defined as subjects with available plasma MMP‐8, PaO_2_/FiO_2_, obesity, and influenza vaccination data. Of the 72 infected subjects with plasma MMP‐8 and PaO_2_/FiO_2_ measurements, 64 had complete covariate data and were included in the adjusted analysis. Because plasma MMP‐8 levels were not normally distributed, a sensitivity analysis was performed using log_10_‐transformed plasma MMP‐8 levels. No global adjustment was applied across different inflammatory mediators and time points because these analyses were exploratory and each mediator was treated as a distinct biological outcome. Accordingly, these findings should be interpreted as hypothesis‐generating.

## 3 Results

### 3.1 Plasma MMP‐8 Levels Are Increased in IAV‐Infected Human Subjects

Table 1 shows the demographic and clinical characteristics of the human cohort. Human subjects with laboratory‐confirmed seasonal IAV or A/California/07/2009 H1N1 infection had ~30‐fold higher plasma MMP‐8 levels than age‐ and sex‐matched healthy controls (Figure 1A). Because the groups differed markedly in obesity prevalence and influenza vaccination status, this between‐group comparison was interpreted as descriptive. In unadjusted analysis, plasma MMP‐8 levels were inversely related to the severity of lung injury as assessed by the PaO_2_/FiO_2_ ratio (Pearson *r* = −0.384, *p* = 0.0009, *n* = 72) (Figure 1B). In a complete‐case analysis of 64 infected subjects with available covariate data, plasma MMP‐8 levels remained significantly associated with lower PaO_2_/FiO_2_ ratios (*β* = −1.138, 95% CI: −1.817 to −0.459, *p* = 0.001, *n* = 64). Similar results were obtained when log_10_‐transformed plasma MMP‐8 levels were used in the model (*β* = −74.18, 95% CI: −119.14 to −29.22, *p* = 0.002, *n* = 64). These findings support an association between plasma MMP‐8 and lung injury severity but do not establish its independent prognostic performance.

**FIGURE 1:**
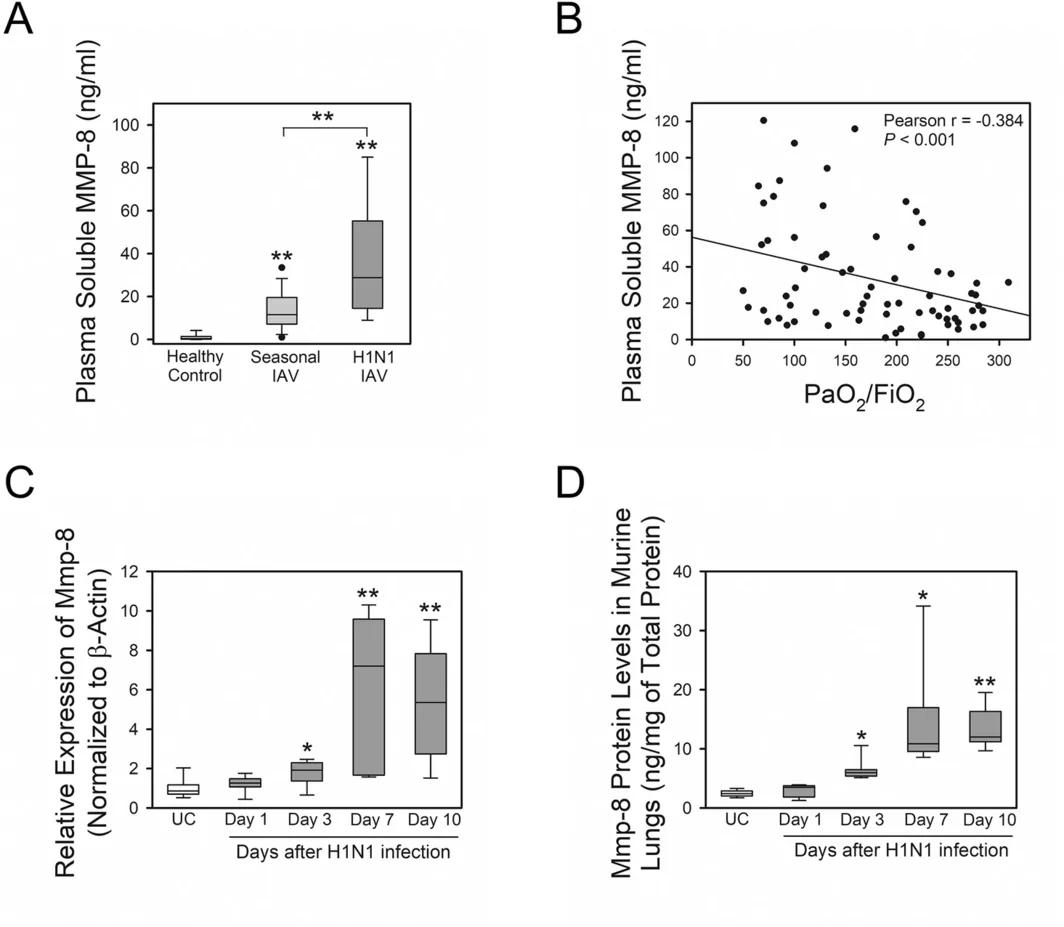
MMP‐8 levels are increased in plasma samples and/or lung samples from IAV‐infected human subjects and WT mice and are inversely associated with the PaO_2_/FiO_2_ ratio in human subjects. (A) MMP‐8 protein levels were measured in plasma samples from 17 healthy controls and 19 or 57 subjects infected with seasonal influenza or A/California/04/2009 H1N1, respectively. (B) Plasma MMP‐8 levels are inversely associated with the PaO_2_/FiO_2_ ratio; *n* = 72 subjects infected with seasonal IAV or H1N1 IAV. Data were analyzed using the Pearson Correlation test; *p* ≤ 0.05 was considered to be statistically significant. (C) *Mmp‐8* mRNA levels were measured in lungs from uninfected WT control (10 mice) and WT mice infected with 10^3^ PFU A/California/04/2009 H1N1 for 1–10 days using real‐time RT‐PCR (7–11 mice/group). (D) MMP‐8 protein levels were measured in lungs from 5 uninfected WT mice and WT mice infected with 10^3^ PFU A/California/04/2009 H1N1 for 1–10 days (5–8 mice/group) using an ELISA. In C and D, boxes in the box plots show the medians and 25th and 75th percentiles, and the whiskers show the 10th and 90th percentiles. Data were analyzed using a Kruskal–Wallis One‐Way ANOVA test followed by pair‐wise testing with Mann–Whitney *U* tests. *p ≤ 0.05; **p ≤ 0.001 versus uninfected control.

### 3.2 *Mmp‐8* Is Upregulated in the Lungs of H1N1‐IAV‐Infected WT Mice

When C57BL/6 WT mice were infected with 10^3^ PFU A/California/04/2009 H1N1 IAV, steady‐state *Mmp‐8* mRNA and MMP‐8 protein levels in lung tissues were both strikingly upregulated on Day 3 p.i., and levels increased further on Days 7 and 10 p.i. (Figure 1C,D). Double immunostaining experiments localized the cellular sources of MMP‐8 in murine lungs. As expected, uninfected WT mice had minimal or no MMP‐8 staining in their lungs (Figure 2). Infected WT mice had increased MMP‐8 staining in airway epithelial cells on Days 3, 7, and 10 p.i. (Figure 2A,B). PMNs were not present in the airways of uninfected mice, but MMP‐8 staining was detected in PMNs recruited to the airways of IAV‐infected mice (Figure 2C,D). The percentage of macrophages staining positively for MMP‐8 in the lungs of WT mice increased over Days 3–10 after H1N1 infection (Figure 2E,F).

**FIGURE 2:**
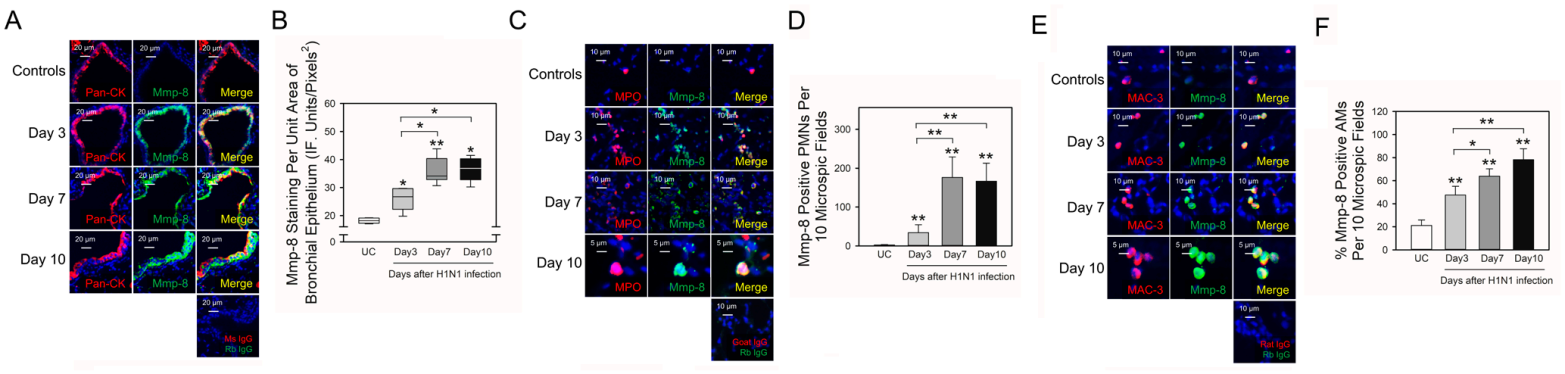
MMP‐8 staining is increased in airway epithelial cells and macrophages and is present in PMNs recruited to the lungs of WT mice infected with H1N1. Lung sections from uninfected control mice and WT mice on Days 3, 7, and 10 after H1N1 infection were analyzed. (A and B) Representative double immunostaining images of MMP‐8 (green) and Pan‐CK (red; marker of lung epithelial cells) (A). MMP‐8 staining was quantified per unit area of bronchial epithelium using MetaMorph software (B). (C and D) Representative double immunostaining images of MMP‐8 (green) and MPO (red; marker of PMNs) (C). The percentage of PMNs staining positively for MMP‐8 was quantified (D). (E and F) Representative double immunostaining images of MMP‐8 (green) and MAC‐3 (red; marker of macrophages) (E). The percentage of macrophages staining positively for MMP‐8 was quantified (F). The lungs of infected mice were also stained with non‐immune isotype‐matched primary antibodies [rabbit (Rb) IgG, murine (Ms) IgG, rat IgG, or goat IgG] and are shown in the lower panels. Nuclei in the sections were counterstained blue using DAPI. Immunostained lung sections were examined with a confocal microscope, and merged images are shown in the right panels. Images shown are representative of sections from 5 to 8 mice per experimental group. No staining was detected in the lungs of infected WT mice stained with a non‐immune primary antibody (lower panels). Boxes in the box‐plots show the median values and 25th and 75th percentiles, and whiskers show the 10th and 90th percentiles. *n* = 5–8 mice per group. Data were analyzed using a Kruskal–Wallis One‐Way ANOVA test followed by pair‐wise testing with Mann–Whitney *U* tests. The percentage of PMNs and alveolar macrophages that were positively stained for MMP‐8 was quantified in 10 randomly acquired microscopic fields (at magnification 200×). The bars show the mean ± SD. Data were analyzed using a One‐Way ANOVA test followed by pair‐wise testing with Student's *t*‐tests. *p ≤ 0.05; **p ≤ 0.001 versus uninfected control or the group indicated.

### 3.3 *Mmp‐8* ^*−/−*^ Mice Are Protected From H1N1 IAV‐Induced Weight Loss and Mortality, and Have a Lower Lung Burden of H1N1 IAV but Greater Lung Injury

Following infection with an LD_80_ inoculum of H1N1 IAV, *Mmp‐8* ^*−/−*^ mice had reduced body weight loss compared with WT mice on Days 4–9 p.i. (Figure 3A) and showed a modest improvement in survival following severe infection (Figure 3B). Compared with H1N1‐infected WT mice, H1N1‐infected *Mmp‐8* ^*−/−*^ mice had lower lung viral titers on Days 3–10 p.i. (Figure 3C) but significantly greater lung injury as assessed by: (1) higher BALF albumin levels (Figure 3D); and (2) respiratory mechanics showing stiffer lungs (as assessed by lower quasi‐static lung compliance and higher tissue and peripheral airway elastance values) on Day 7 p.i. (Figure 3E,F).

**FIGURE 3:**
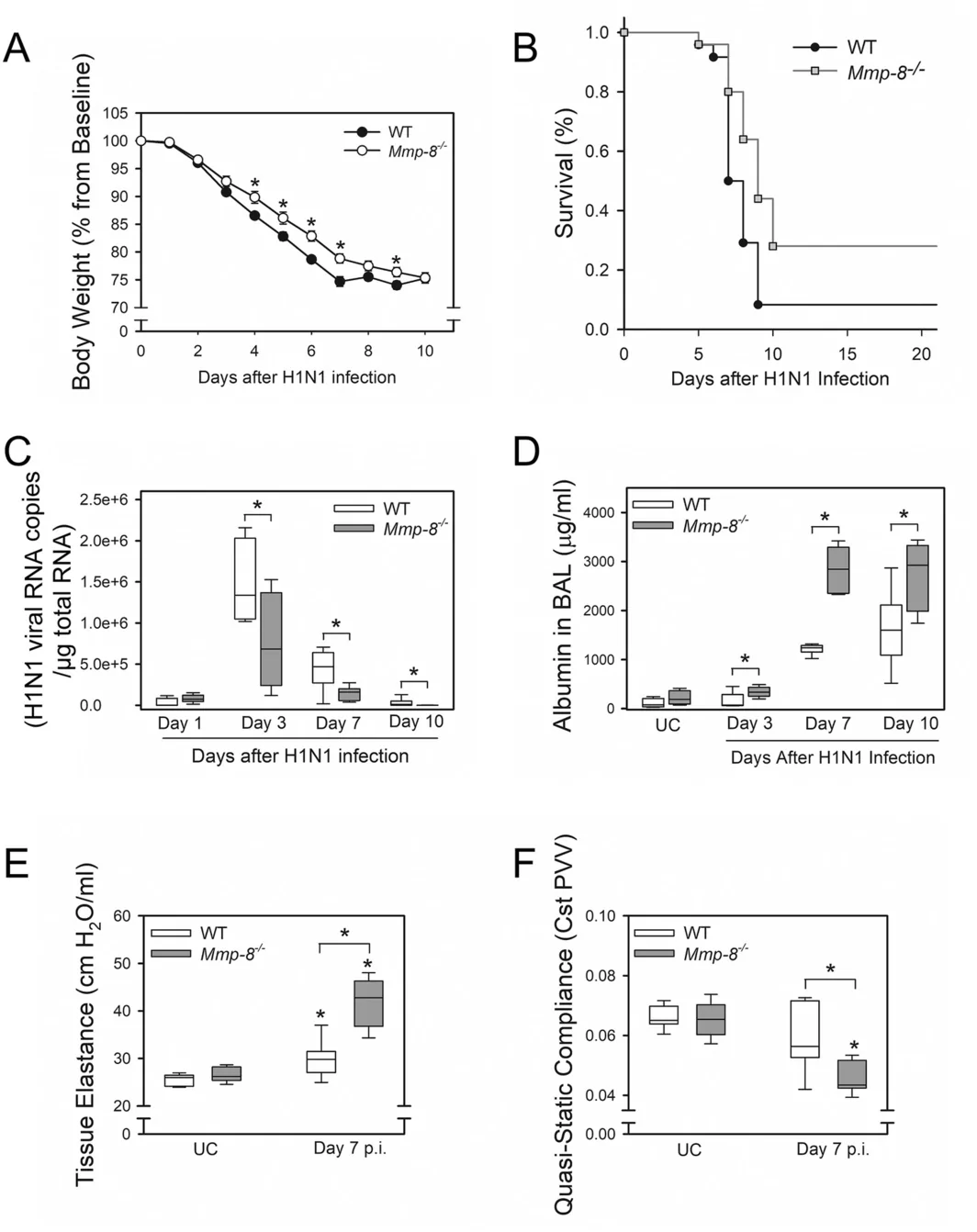
*Mmp‐8* ^*−/−*^ mice are protected from H1N1‐induced weight loss and mortality, and have a lower lung burden of H1N1 IAV, but greater lung injury. In A and B, 24 WT and 25 *Mmp‐8* ^*−/−*^ mice were infected with 10^4^ PFU H1N1. (A) Changes in body weight as a % of baseline body weight were recorded daily for the first 10 days p.i. Data are mean ± SEM; *p ≤ 0.05 versus WT mice at the same time point. (B) Survival was monitored twice daily, and Kaplan–Meier survival plots were generated. Gehan‐Breslow analysis confirmed that there was a significant difference between the survival curves (*p* = 0.006). In (C–F) WT and *Mmp‐8* ^*−/−*^ mice were infected with an LD_20_ inoculum of H1N1 IAV (10^3^ PFU) to permit tissue harvest for assessment of lung viral titers, BALF albumin levels, and respiratory mechanics. (C) H1N1 viral RNA copy numbers were measured in lung homogenates by quantitative real‐time RT‐PCR and expressed as viral RNA copies per μg of total lung RNA (*n* = 6–9 mice per group). (D) Albumin levels were measured in BAL samples from H1N1‐infected WT and *Mmp‐8* ^*−/−*^ mice (5–11 mice per group). (E and F) WT and *Mmp‐8* ^*−/−*^ mice were infected with H1N1 (8–9 mice per group), and respiratory mechanics (tissue elastance in E and quasi‐static compliance in F) were measured using a small animal ventilator. In C–F, boxes in the box plots show the median values and 25th and 75th percentiles, and whiskers show the 10th and 90th percentiles. Data were analyzed using a Kruskal–Wallis One‐Way ANOVA test followed by pair‐wise testing with Mann–Whitney *U* tests. *p ≤ 0.05 versus uninfected mice belonging to the same genotype or the group indicated.

### 3.4 H1N1 IAV‐Infected *Mmp‐8* ^*−/−*^ Mice Have Greater Pulmonary Necroptosis Than WT Mice

The expression of 84 genes involved in TLR‐mediated signal transduction and innate immunity was evaluated using pooled RNA from murine whole‐lung samples. Among these genes, six showed greater than three‐fold upregulation in pooled lung RNA from infected *Mmp‐8* ^*−/−*^ mice relative to WT mice at one or more time points and were selected for descriptive presentation in Table 2. Because the array was performed using pooled RNA without independent biological replicates, these findings were interpreted descriptively. The complete heatmap containing all 84 genes, including genes with increased or decreased expression, is provided in Figure 4A. Among the genes meeting the descriptive screening threshold, *Eif2ak2* (*Pkr*) showed the largest increase and was subsequently evaluated by qRT‐PCR using individual lung samples with independent biological replicates (Figure 4B). PKR is an RNA‐responsive kinase involved in IFN‐α/β‐induced necroptosis [29]. Necroptosis is a programmed form of pro‐inflammatory cell death in which virus‐infected cells undergo caspase‐independent “cellular suicide” in the presence of viral inhibitors of intracellular caspases. The release of cellular contents by infected cells triggers inflammation and an immune response to the virus that limits viral spreading [30, 31]. Necroptosis can be induced by Type I IFN signaling [29]. IFN‐α/β signaling upregulates *Pkr* expression, which stimulates receptor‐interacting protein kinases‐1 and ‐3 (RIP1‐RIP3) complex formation, leading to the formation of the “necrosome” complex, which induces cellular lysis. This process releases pro‐inflammatory damage‐associated molecular patterns (DAMPs) and exposes viral particles to the immune system, leading to an adaptive immune response to the virus [30, 31, 32].

**TABLE 2:** Genes up‐regulated in *Mmp‐8* ^*−/−*^ mice lung tissues after IAV infection.

Gene	Day 1 p.i. (fold change over WT)	Day 3 p.i. (fold change over WT)	Day 7 p.i. (fold change over WT)
Eif2ak2 (Pkr)	5.6	7.2	0.8
G‐csf	3.5	2.2	0.8
Il‐12α	4.1	4.7	0.6
Il‐1α	3.1	0.7	0.6
Il‐1β	5.5	3.1	2
Il‐6	3.1	3.8	1.5

*Note:* WT and *Mmp‐8* ^*−/−*^ mice were infected with 10^3^ PFU H1N1 by the intranasal route. Total RNA was isolated from the whole lung tissues on Days 1, 3 and 7 post infection (p.i.). For each experimental group and time point, equal amounts of lung RNA from 6 to 8 mice were pooled before analysis using the RT^2^ Profiler Mouse TLR Signaling Pathway PCR Array. Fold changes represent gene expression in pooled lung RNA from infected *Mmp‐8* ^*−/−*^ mice relative to pooled lung RNA from infected WT mice at the corresponding time point. Because each group and time point was represented by a single pooled measurement without independent biological replicates, inferential statistical testing and FDR correction were not applicable. Genes showing greater than three‐fold upregulation at one or more time points were selected for descriptive presentation. The three‐fold threshold was used as an exploratory screening criterion to identify genes with the largest expression differences and does not indicate statistical significance.

**FIGURE 4:**
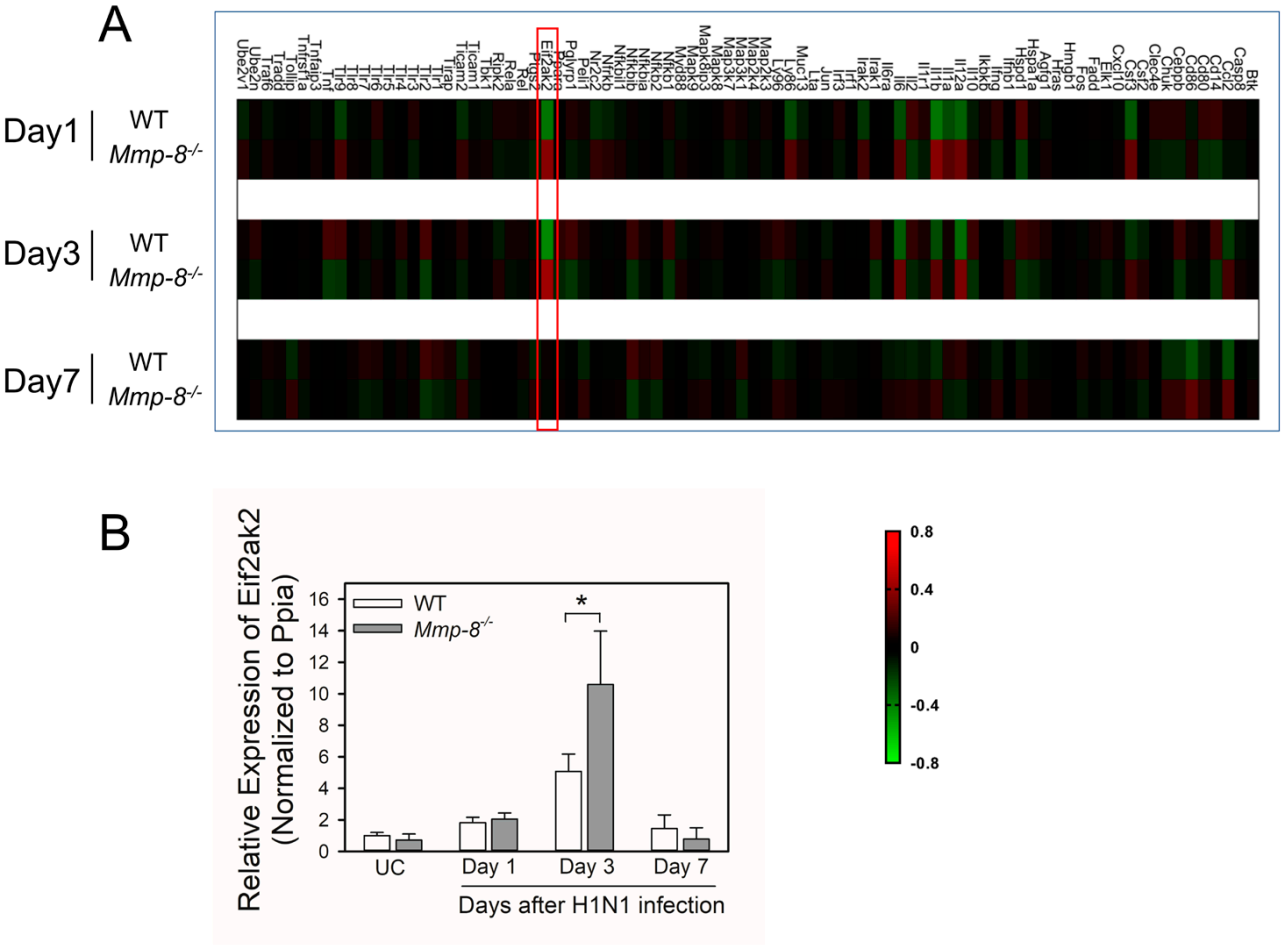
H1N1 IAV‐infected *Mmp‐8* ^*−/−*^ mice have increased expression of genes involved in TLR‐mediated signal transduction and innate immunity in lung tissue. (A) For each experimental group and time point, equal amounts of lung RNA from 6 to 8 mice were pooled before analysis using the RT^2^ Profiler Mouse TLR Signaling Pathway PCR Array. The heatmap shows the expression patterns of all 84 genes evaluated in pooled lung RNA. Intensity values were log_10_‐transformed and centered to the mean expression value for each gene across Days 1, 3, and 7 p.i. Red and green indicate relatively higher and lower expression, respectively. In (B) *Eif2ak2* (*Pkr*) mRNA levels were measured in lungs from 5 to 7 uninfected mice and 6–8 mice infected with 10^3^ PFU H1N1 for 1, 3, or 7 days using RT‐PCR. The bars show the mean ± SD. Data were analyzed using a One‐Way ANOVA test followed by pair‐wise testing with Student's *t*‐tests. *p ≤ 0.05 versus the group indicated.

Double immunofluorescence staining demonstrated increased levels of the activated (phosphorylated) forms of RIP3 (phospho‐RIP3‐Ser^232^, Figure 5A,B) and its downstream effector MLKL (p‐MLKL‐Ser^345^, Figure 5C,D) in airway epithelial cells from infected *Mmp‐8* ^*−/−*^ mice compared with WT controls on Day 3 p.i. Consistently, whole‐lung levels of phosphorylated RIP3 and phosphorylated MLKL (Figure 5E–G) were also elevated in infected *Mmp‐8* ^*−/−*^ mice relative to WT mice on Day 3 p.i. In contrast, steady‐state Rip1 and *Rip3* mRNA expression (data not shown), as well as total lung RIP3 and MLKL protein levels (Figure 5E), were comparable between infected *Mmp‐8* ^*−/−*^ and WT mice. These findings demonstrate increased activation of the necroptotic signaling pathway in airway epithelial cells of H1N1‐infected *Mmp‐8* ^*−/−*^ mice compared with WT mice. Furthermore, Necrostatin‐1 (Nec‐1) treatment significantly reduced BAL albumin levels in H1N1‐infected *Mmp‐8* ^*−/−*^ mice, indicating attenuated lung injury (Figure 5H). In contrast, Nec‐1 significantly increased lung viral titers (Figure 5I), suggesting impaired viral clearance. These findings provide functional evidence that RIP1‐dependent necroptotic signaling contributes to both lung injury and antiviral defense during influenza infection in *Mmp‐8* ^*−/−*^ mice.

**FIGURE 5:**
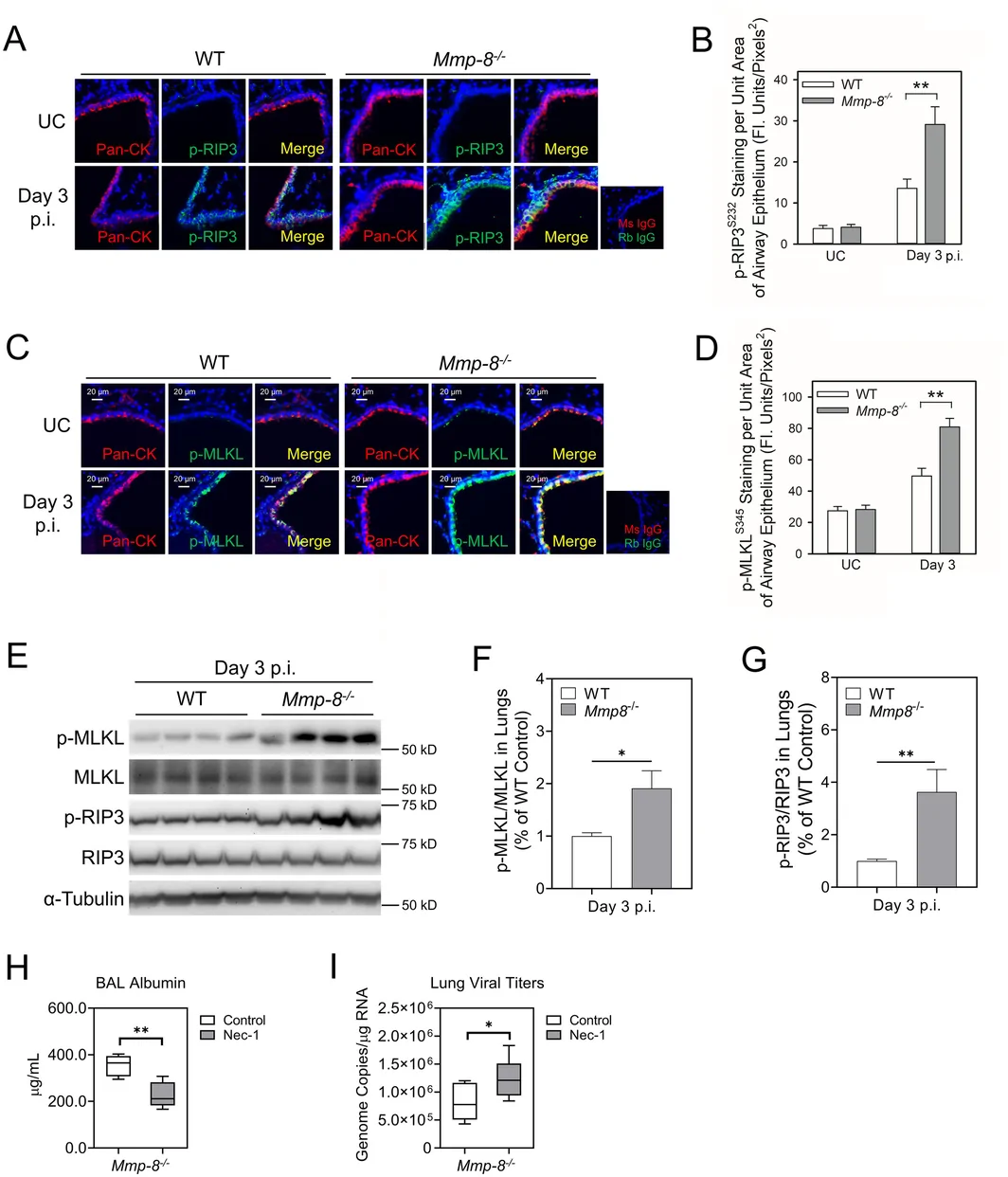
H1N1 IAV infection induced greater necroptosis in airway epithelial cells in *Mmp‐8* ^*−/−*^ versus WT mice. WT and *Mmp‐8* ^*−/−*^ mice were infected with H1N1 via the intranasal route, and at intervals post infection, lungs were inflated and removed. Lung sections were double immunostained with a red fluorophore for a marker of lung epithelial cells (Pan‐CK) and with a green fluorophore for p‐RIP3‐Ser^232^ (A) or p‐MLKL‐Ser^345^ (C). Lungs were also stained with a non‐immune rabbit (Rb) IgG and a non‐immune mouse (Ms) IgG, as shown (lower panels). DAPI was used to counterstain the nuclei. Lung sections were analyzed using a confocal microscope, and merged images are shown in A and C. The images shown are representative of 5–7 mice per group. In B and D, p‐RIP3‐Ser^232^ staining (B) and p‐MLKL‐Ser^345^ staining (D) in airway epithelial cells were quantified in 10 randomly acquired images per lung section using MetaMorph software, and the staining signal was normalized to the unit area of bronchial wall evaluated. The bars show the mean ± SD; *n* = 5–7 mice per group. Data were analyzed using a One‐Way ANOVA test followed by pair‐wise testing with Student's *t*‐tests. **p ≤ 0.001 versus the group indicated. In E–G, WT and *Mmp‐8* ^*−/−*^ mice were infected with H1N1 via the intranasal route. On Day 3 p.i., p‐MLKL‐Ser^345^, total MLKL, p‐RIP3‐Ser^232^, total RIP3, and housekeeping control (α‐Tubulin) were quantified in homogenates of lung samples using Western blotting and densitometry. The p‐MLKL‐Ser^345^ or p‐RIP3‐Ser^232^ signals were normalized to total MLKL or RIP3 levels and expressed as a % of the values for uninfected WT mice. The images shown in (E) are representative of 4 mice per group. (F and G) The bars show means ± SD; *n* = 6 mice/group. (H and I) H1N1‐infected *Mmp‐8* ^*−/−*^ mice were treated with the RIP1 inhibitor Necrostatin‐1 (Nec‐1) or vehicle containing 1% DMSO in saline beginning 24 h after infection. BAL fluid albumin levels (H) and lung viral titers (I) were measured. Data are presented as mean ± SD or box plots as indicated. *p ≤ 0.05; **p ≤ 0.01 versus the indicated group.

### 3.5 H1N1 IAV‐Infected *Mmp‐8* ^*−/−*^ Mice Have Increased Pulmonary Type I IFN and Th1 Immune Responses

Type I IFNs serve as a major host defense pathway against IAV infection [33]. IAV RNA is recognized as foreign by pattern recognition receptors (PRRs), which trigger the secretion of Type I IFNs [34]. Type I IFNs limit further viral growth and spread to uninfected host cells by inducing necroptosis of infected cells [29] and inducing the expression of antiviral proteins by cells susceptible to viral infection [35]. Infected *Mmp‐8* ^*−/−*^ mice had higher lung protein levels of IFN‐α on Day 3 p.i. (Figure 6A), IFN‐β on Days 3 and 7 p.i. (Figure 6B), but similar levels of IFN‐γ (a Type II IFN; Figure 6C) at all time points tested. However, steady‐state mRNA levels of *Ifn‐α*, *Ifn‐β*, and *Ifn‐γ* in whole lung samples were similar in infected *Mmp‐8* ^*−/−*^ vs. WT mice (Figure S1), indicating that the higher IFN‐α and IFN‐β lung protein levels did not reflect differences in transcript levels in H1N1‐infected *Mmp‐8* ^*−/−*^ vs. WT mice. Consistent with enhanced Type I IFN signaling, IFN‐β treatment significantly increased *Eif2ak2* (*Pkr*) expression and immunofluorescence signals for phosphorylated RIP3 and MLKL in primary airway epithelial cells from both WT and *Mmp‐8* ^*−/−*^ mice (Figure 6D–H). However, no genotype‐dependent differences were detected, suggesting that *Mmp‐8* deficiency does not substantially alter the intrinsic responsiveness of airway epithelial cells to IFN‐β‐induced necroptotic signaling under these experimental conditions.

**FIGURE 6:**
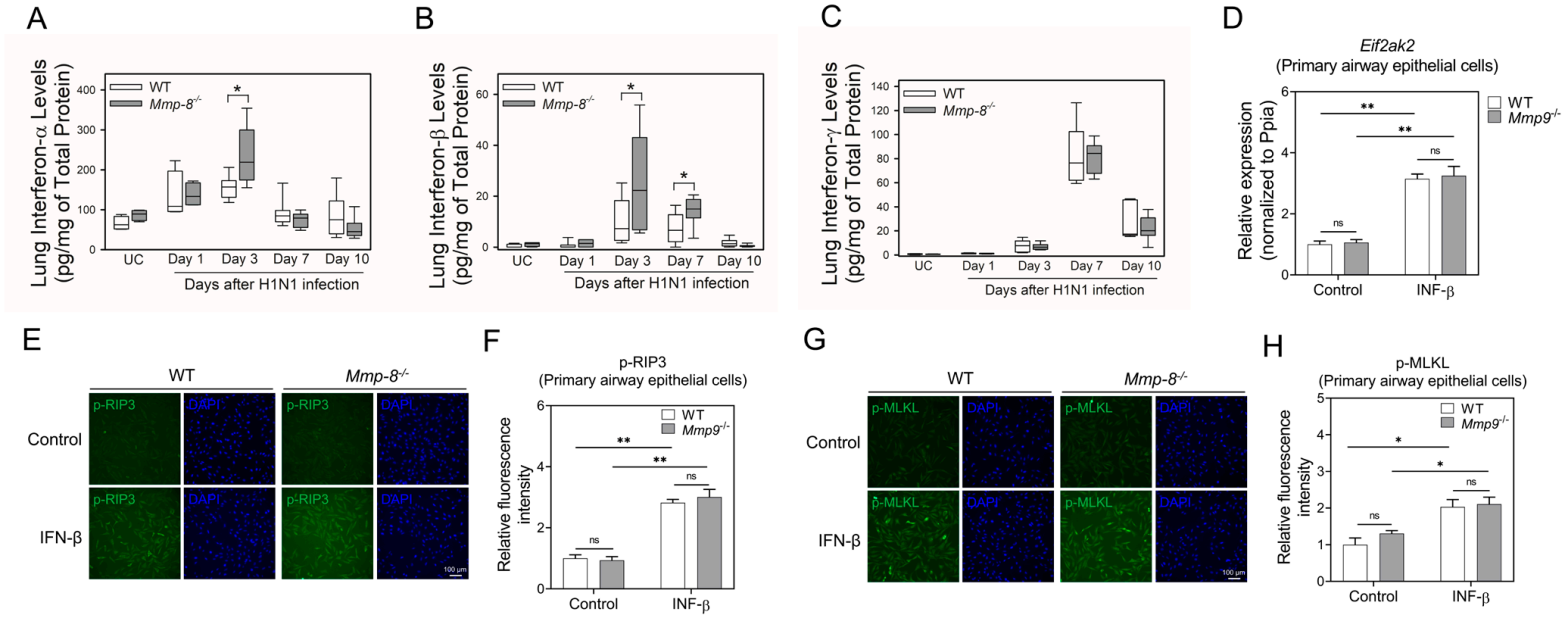
H1N1 IAV infection induces greater increases in Type I (but not Type II) IFN levels in *Mmp‐8* ^*−/−*^ versus WT mice. WT and *Mmp‐8* ^*−/−*^ mice were infected with 10^3^ PFU H1N1 via the intranasal route. Uninfected mice were used as controls. At intervals post‐infection, lung levels of IFN‐α (A, *n* = 4–10 mice per group), IFN‐β (B, *n* = 4–15 mice per group), and IFN‐γ (C, *n* = 4–10 mice per group) were measured using ELISAs and normalized to lung total protein levels. (D–H) Primary airway epithelial cells isolated from WT and *Mmp‐8* ^*−/−*^ mice were treated with 20 ng/mL rhIFN‐β for 24 h. *Eif2ak2* (*Pkr*) mRNA expression was measured by RT‐qPCR (D). Representative immunofluorescence images are shown in E and G, and the corresponding p‐RIP3 and p‐MLKL signals are quantified in F and H, respectively. In A–C, boxes in the box‐plots show the medians and 25th and 75th percentiles, and the whiskers show the 10th and 90th percentiles. Data were analyzed using a Kruskal–Wallis One‐Way ANOVA followed by pair‐wise testing with Mann–Whitney *U* tests. In D–H, data are from 3 independent experiments and are presented as mean ± SD. *p ≤ 0.05; **p ≤ 0.01 versus the indicated group.

In the pooled‐RNA PCR array, *Il‐1α*, *Il‐1β*, *Il‐6*, and *Il‐12α* showed increased expression in infected *Mmp‐8* ^*−/−*^ lungs relative to WT lungs at one or more time points (Table 2). Pulmonary innate and adaptive immune responses protect experimental animals from IAV‐induced lung disease by killing IAV‐infected host cells [36]. Thus, leukocyte counts were measured in lung samples from IAV‐infected mice. H1N1‐infected *Mmp‐8* ^*−/−*^ mice had higher BAL lymphocyte counts (Figure 7A) but similar BAL total leukocyte (Figure S2A), macrophage (Figure S2B), and PMN counts (Figure S2C) compared with WT mice. When immune cell subsets were quantified using immunostaining and flow cytometry, H1N1‐infected WT and *Mmp‐8* ^*−/−*^ mice had similar percentages of BAL PMNs (Figure S2D), macrophages (Figure S2E), and NK cells (Figure S2F). However, *Mmp‐8* ^*−/−*^ mice had higher frequencies of BAL macrophages expressing MHC Class II molecules (which participate in antigen presentation; Figure 7B), CD4^+^ T cells (Figure 7C), and CD8^+^ T cells (Figure 7D) than WT mice on Day 7 p.i.

**FIGURE 7:**
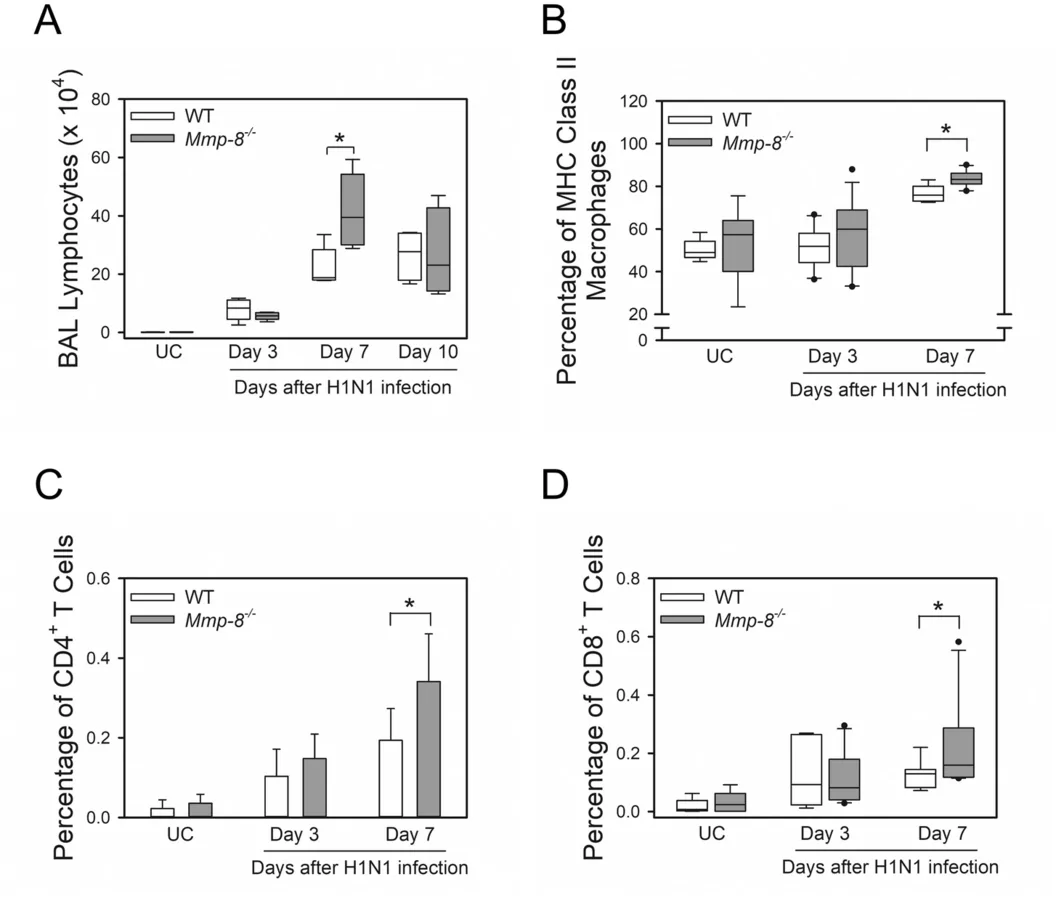
H1N1 IAV‐infected *Mmp‐8* ^*−/−*^ mice have higher BAL lymphocyte, MHC Class II‐expressing macrophage, CD4^+^ T cell, and CD8^+^ T cell counts than WT mice. WT and *Mmp‐8* ^*−/−*^ mice (*n* = 4–10 mice per group) were infected with H1N1 via the i.n. route, or uninfected as controls. BAL was performed on the mice at intervals post‐infection. Lymphocytes (A) were counted in BAL samples. BAL leukocytes were immunostained for MHC Class II‐expressing macrophages (B), CD4^+^ T cells (C), CD8^+^ T cells (D), and analyzed by flow cytometry. In A, B and D, the boxes in the box‐plots show the medians and 25th and 75th percentiles, and the whiskers show the 10th and 90th percentiles. Data were analyzed using a Kruskal–Wallis One‐Way ANOVA test followed by pair‐wise testing with Mann–Whitney *U* tests. *p ≤ 0.05 versus the group indicated. In C, the bars show means ± SD. Data were analyzed using a One‐Way ANOVA test followed by pair‐wise testing with Student's *t*‐tests. *p ≤ 0.05 versus the group indicated.

Lung levels of cytokines and chemokines that regulate the recruitment, activation, and survival of leukocytes (CCL‐2, CCL‐3, CCL‐5, CXCL‐1, CXCL‐2, CXCL‐6, TNF‐α, IL‐6, IL‐1β, IL‐17, GM‐CSF, M‐CSF, and G‐CSF), and anti‐inflammatory mediators (IL‐10 and TGF‐β) were measured in WT and *Mmp‐8* ^*−/−*^ mice whole lung homogenates after H1N1 infection. Levels of CCL‐3, CXCL‐2, GM‐CSF, M‐CSF, IL‐17, TNF‐α, IL‐10, and TGF‐β in lung homogenates were similar in IAV‐infected WT and *Mmp‐8* ^*−/−*^ mice (Figure S3). However, lung levels of IL‐1β, IL‐6, CCL‐2, CXCL‐1, CXCL‐6, and G‐CSF were higher in IAV‐infected *Mmp‐8* ^*−/−*^ mice than in WT mice on Day 1 and/or Day 3 p.i. (Figure 8A–F). Levels of CCL‐5 were higher in H1N1 IAV‐infected *Mmp‐8* ^*−/−*^ versus WT mice on Days 7 and 10 p.i. (Figure 8G).

**FIGURE 8:**
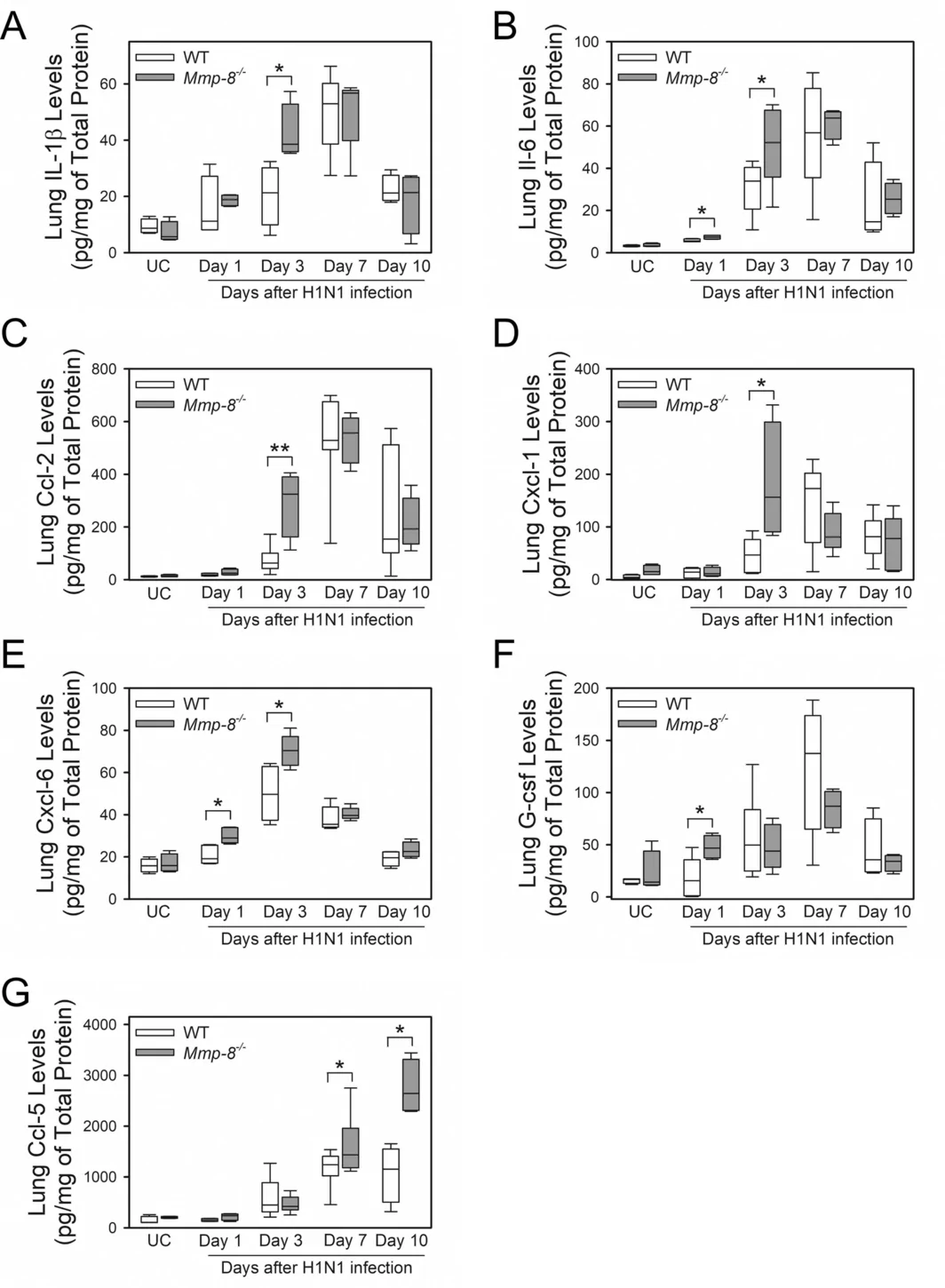
H1N1 IAV‐infected *Mmp‐8* ^*−/−*^ mice have increased lung levels of some pro‐inflammatory mediators. WT and *Mmp‐8* ^*−/−*^ mice (4–10 mice/group) were infected with H1N1 IAV. Lung levels of IL‐1β (A; *n* = 4–5 mice per group), IL‐6 (B; *n* = 4–10 mice per group), CCL‐2 (C; *n* = 4–10 mice per group), CXCL‐1 (D; *n* = 4–10 mice per group), CXCL‐6 (E; *n* = 4–8 mice per group), G‐CSF (F; *n* = 4–10 mice per group) and CCL‐5 (G; *n* = 4–10 mice per group) were measured at intervals p.i. using ELISAs and normalized to lung total protein levels. In A–G, boxes in the box‐plots show the median values and 25th and 75th percentiles, and whiskers show the 10th and 90th percentiles. Data were analyzed using a Kruskal–Wallis One‐Way ANOVA test followed by pair‐wise testing with Mann–Whitney *U* tests. *p ≤ 0.05; **p ≤ 0.001 versus the group indicated.

### 3.6 MMP‐8 Cleaves Some Pro‐Inflammatory Mediators In Vitro

MMP‐8 has an active metalloproteinase domain and cleaves and inactivates CCL‐3 during ALI in mice [6]. MMP‐9 proteolytically inactivates IFN‐β in vitro [37]. Thus, we investigated whether MMP‐8 degrades Type I IFNs and cytokines that were elevated in the lungs of H1N1 IAV‐infected *Mmp‐8* ^*−/−*^ mice. When tested in vitro, active human MMP‐8 produced only modest cleavage of IFN‐α2 and IFN‐β, as assessed by the appearance of a small quantity of a lower molecular mass cleavage product. Generation of this product was blocked when a small molecule metalloproteinase inhibitor was added to the reactions, confirming that the active site of MMP‐8 is required for these activities (Figure 9A,B). Thus, the higher IFN‐α/β‐mediated antiviral immunity in H1N1 IAV‐infected *Mmp‐8* ^*−/−*^ lungs is unlikely to be due to the lack of *Mmp‐8*‐mediated cleavage of IFN‐α/β.

**FIGURE 9:**
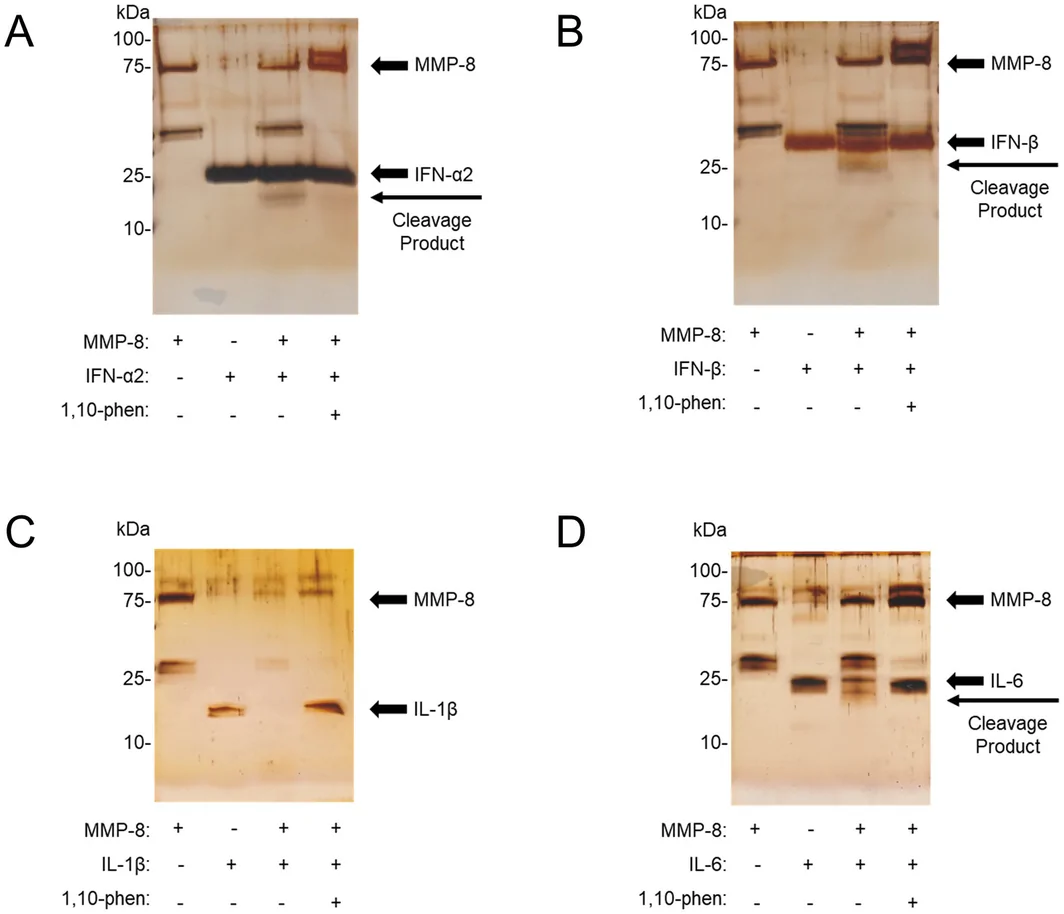
Active MMP‐8 cleaves IL‐1β and IL‐6 in vitro. Recombinant human (rh) pro‐MMP‐8 was activated, and its activity was confirmed in each experiment using a quenched fluorogenic substrate that is susceptible to cleavage by MMP‐8, as described in the Supporting Information. Recombinant human cytokines, including 1000 nM IFN‐α2 (A), 1000 nM IFN‐β (B), 1100 nM IL‐1β (C), and 900 nM IL‐6 (D), were incubated with or without 300 nM active rhMMP‐8 for 18 h, with or without a non‐selective metalloproteinase inhibitor (1,10‐phenanthroline; 1,10 phen), which inhibits MMP‐8. The reaction products were separated on Tris‐tricine gels and visualized with silver staining. The arrows indicate the bands corresponding to rhMMP‐8 and the intact cytokines. The long arrows indicate the cleavage products of IFN‐α2, IFN‐β, and IL‐6 (in A, B, and D). MMP‐8 completely degraded IL‐1β; no degradation products were detected (C). The addition of 1,10‐phenanthroline blocked the cleavage or degradation of the four cytokines that was mediated by active MMP‐8.

The generation of IL‐1β by the inflammasome promotes host defense against IAV infection [38]. Active human MMP‐8 completely degraded IL‐1β (Figure 9C) and substantially degraded IL‐6 (Figure 9D) in vitro. MMP‐8‐mediated degradation of IL‐1β and IL‐6 was completely inhibited by adding a metalloproteinase inhibitor to the reaction mixtures. Thus, the biochemical capacity of MMP‐8 to cleave IL‐1β and IL‐6 in vitro raises the possibility that loss of this activity contributes to their elevated lung levels in infected *Mmp‐8* ^*−/−*^ mice; however, this mechanism was not established in vivo. In contrast, active human MMP‐8 did not degrade human CCL‐2, CCL‐5, or IL‐8 (the human ortholog of Cxcl‐1) (data not shown). Thus, the increased levels of several pro‐inflammatory mediators may contribute to the enhanced immune responses observed in infected *Mmp‐8* ^*−/−*^ mice, although the mechanisms responsible for their accumulation remain uncertain.

## 4 Discussion

Our data identify MMP‐8 as a host‐response regulator during IAV infections. We report for the first time that plasma MMP‐8 levels are substantially increased in human subjects infected with pandemic H1N1 and seasonal IAV, and that levels inversely correlate with the PaO_2_/FiO_2_ ratio, a clinical indicator of ALI. MMP‐8 expression was significantly elevated in airway epithelial cells, macrophages, and PMNs following H1N1 infection in WT mice. Notably, H1N1‐infected *Mmp‐8* ^*−/−*^ mice exhibited lower mortality and lung viral loads compared with WT controls, despite experiencing more severe lung injury.

The reduced viral burdens in *Mmp‐8* ^*−/−*^ mice were accompanied by increased pulmonary Type I IFN levels, enhanced necroptotic signaling in airway epithelial cells, and augmented adaptive immune responses. These pathways may contribute to more efficient viral clearance, although their relative contributions remain uncertain. However, the mechanism linking *Mmp‐8* deficiency to increased pulmonary Type I IFN protein levels remains unclear and represents a key unresolved question of this study. The increased pulmonary adaptive immune response in IAV‐infected *Mmp‐8* ^*−/−*^ mice may be driven, at least in part, by increased necroptosis, which can promote extracellular release of viral particles and DAMPs that enhance immune activation [30, 31, 32]. In addition, H1N1‐infected *Mmp‐8* ^*−/−*^ mice exhibited higher pulmonary levels of the pro‐inflammatory cytokines IL‐1β and IL‐6, both of which have been implicated in promoting effective host immune responses to IAV [39, 40]. Our in vitro findings raise the possibility that the increased levels of IL‐1β and IL‐6 in infected *Mmp‐8* ^*−/−*^ mice may partly reflect the loss of MMP‐8‐mediated proteolytic processing. However, this possibility was not directly tested in vivo.

Prior studies report that the expression of MMP‐14, ‐9, and ‐13 is increased in IAV‐infected cells and that antibody‐mediated inhibition of MMP‐14 or deficiency of *Mmp‐9* protects mice from IAV‐mediated disease [18, 19, 41, 42]. However, the contribution of MMP‐8 to IAV‐induced disease pathogenesis has not been investigated previously. We report that plasma MMP‐8 levels were > 30‐fold higher in human subjects infected with H1N1 and seasonal IAV than in uninfected controls. Elevated plasma MMP‐8 levels likely reflect increased activation and degranulation of circulating PMNs, as MMP‐8 is predominantly expressed by PMNs [6], and circulating PMNs are activated in humans infected with IAV [21, 43]. Furthermore, the inverse association between plasma MMP‐8 levels and the PaO_2_/FiO_2_ ratio suggests that plasma MMP‐8 may have potential value as a candidate marker of lung injury severity during IAV infection. However, its clinical and prognostic value requires validation in larger prospective cohorts with longitudinal outcomes.

In the mouse model, pulmonary *Mmp‐8* mRNA and MMP‐8 protein levels were increased as early as Day 3 p.i., with airway PMNs, macrophages, and epithelial cells serving as the main cellular sources of MMP‐8. The early rise in MMP‐8 likely results from the rapid recruitment of PMNs to the infected lung [8], whereas the sustained increase observed on Days 7–10 p.i. is likely driven by enhanced *Mmp‐8* expression in activated airway macrophages and epithelial cells [9, 10].

IAV is tropic for airway epithelial cells, and replication of the virus within these cells causes epithelial death and pulmonary inflammation. The lower lung viral burdens of IAV‐infected *Mmp‐8* ^*−/−*^ versus WT mice are likely due to their higher lung levels of Type I IFNs, as signaling via the Type I IFN receptor limits further viral growth and spreading by increasing cellular expression of antiviral proteins, which reduce viral replication [35]. In addition, Type I IFNs also upregulate *Pkr* expression, which promotes RIP1/RIP3 complex formation and the induction of necroptosis [29]. Therefore, the increased pulmonary Type I IFN levels in IAV‐infected *Mmp‐8* ^*−/−*^ mice may contribute to the enhanced necroptotic signaling observed in airway epithelial cells. However, the present data do not establish Type I IFNs as the sole cause of enhanced necroptosis or determine the extent to which this pathway contributes to reduced viral replication [29]. The increased necroptosis in IAV‐infected *Mmp‐8* ^*−/−*^ mice may also have contributed to their greater pulmonary adaptive immune responses. Necroptotic epithelial cells release DAMPs and viral particles, which promote the activation of innate immune cells and enhance subsequent adaptive immune responses.

Consistent with this mechanism, IFN‐β induced *Pkr* expression and increased RIP3 and MLKL phosphorylation in primary airway epithelial cells. However, these cells from WT and *Mmp‐8* ^*−/−*^ mice responded similarly to IFN‐β stimulation, indicating that *Mmp‐8* deficiency does not intrinsically alter epithelial responsiveness to Type I IFNs. Thus, the enhanced necroptotic signaling observed in vivo is associated with increased pulmonary Type I IFN levels and may depend on factors within the lung microenvironment, rather than an intrinsic difference in epithelial responsiveness to IFN‐β.

We initially hypothesized that MMP‐8 cleaves and degrades IFN‐α/β, as MMP‐8 cleaves several cytokines in other disease models [6]. Under this model, the absence of *Mmp‐8*‐mediated cleavage would be expected to increase pulmonary IFN‐α/β levels in infected *Mmp‐8* ^*−/−*^ mice. Surprisingly, human MMP‐8 only modestly cleaved IFN‐α2 and IFN‐β, indicating that the enhanced IFN‐α/β‐mediated antiviral immunity in H1N1 IAV‐infected *Mmp‐8* ^*−/−*^ lungs is unlikely to result from the loss of direct MMP‐8‐dependent cleavage of IFN‐α/β. The mechanism linking MMP‐8 to pulmonary Type I IFN protein levels remains unresolved. Future studies examining the cellular sources of Type I IFNs, upstream IFN‐regulatory pathways, and microenvironment‐dependent mechanisms will be required to define this relationship.

IAV‐infected *Mmp‐8* ^−*/*−^ mice had a greater immune response to IAV as assessed by higher total lymphocyte and CD4^+^ and CD8^+^ T cell counts and enhanced markers of macrophage activation. The augmented adaptive immune response may be driven by increased airway epithelial necroptosis as well as elevated lung levels of CCL‐2, ‐3, and ‐5 and IL‐1α, ‐1β, ‐6, and ‐12. CCL‐2, ‐3, and ‐5 promote the recruitment and activation of monocytes and lymphocytes in the lung, whereas IL‐1α, ‐1β, ‐6, and ‐12 enhance T cell activation. Notably, IL‐1β and IL‐6 have established protective roles during IAV infection through their ability to stimulate immune responses [34, 40, 44]. IL‐1β promotes dendritic cell (DC) CC‐chemokine receptor‐7 expression and migration to draining lymph nodes, thereby enhancing T cell priming [34]. It also directly promotes T cell activation by increasing Il‐2 receptor expression and, together with IL‐6, induces the production of IL‐2 and other T cell growth factors. In addition, IL‐1β activates B cells by inducing IL‐4 expression [44]. IL‐6 further contributes to antiviral immunity by activating CD4^+^ T cells and promoting the proliferation, differentiation, and cytotoxic activity of CD8^+^ T cells during IAV infection [34, 40].

MMP‐8 has been implicated in several inflammatory pulmonary diseases through its ability to cleave and regulate the activity of pro‐inflammatory mediators [5, 6, 7]. Our study demonstrates that MMP‐8 has the biochemical capacity to cleave IL‐1β and IL‐6 in vitro. This finding raises the possibility that loss of MMP‐8‐mediated processing contributes to the elevated lung levels of these cytokines in infected *Mmp‐8* ^*−/−*^ mice. However, the cleavage assays used recombinant proteins at relatively high concentrations and an extended incubation time and did not reproduce the dynamic, protease‐rich environment of the infected lung. Moreover, we did not measure local active MMP‐8 concentrations or detect intact cytokines and MMP‐8‐generated cleavage products in lung tissues. Therefore, the physiological relevance of this cleavage activity remains to be established in vivo. Several inflammatory mediators were elevated in *Mmp‐8* ^*−/−*^ lungs primarily during the early phase of infection, on Days 1 and/or 3, and many returned toward WT levels at later time points. In contrast, CCL‐5 was elevated on Days 7 and 10, suggesting that the inflammatory consequences of *Mmp‐8* deficiency vary over the course of infection. However, the mechanisms and biological consequences of these temporal patterns require further investigation.

Although H1N1‐infected *Mmp‐8* ^*−/−*^ mice had lower IAV‐induced mortality and lung viral burdens, they paradoxically developed more severe ALI. The increased epithelial necroptosis in IAV‐infected *Mmp‐8* ^*−/−*^ mice likely contributed to this heightened lung injury, as necroptotic cell death is a well‐established driver of tissue damage and organ injury [32, 45]. In addition, the augmented immune responses in infected *Mmp‐8* ^*−/−*^ mice may have further exacerbated ALI, given the recognized role of immune cell‐mediated inflammation in the pathogenesis of IAV‐induced ALI [21, 46]. Thus, while the enhanced antiviral and immune responses associated with *Mmp‐8* deficiency improve viral control and survival, they may simultaneously promote immunopathology and lung tissue damage.

The phenotype of H1N1‐infected *Mmp‐8* ^*−/−*^ mice differs substantially from that of *Mmp‐9* ^*−/−*^ mice reported in our previous study [19]. *Mmp‐9* deficiency protected mice from severe H1N1 IAV infection and was associated with reduced mortality, lower viral burdens, reduced E‐cadherin shedding, attenuated myeloid inflammation, and enhanced adaptive immune responses [19]. In contrast, *Mmp‐8* deficiency reduced lung viral burdens and body weight loss but exacerbated lung injury. These divergent phenotypes suggest that MMP‐8 and MMP‐9 perform distinct, nonredundant functions during IAV infection. Mechanistically, MMP‐9 promotes epithelial barrier disruption, at least in part through E‐cadherin shedding, whereas MMP‐8 may influence inflammatory cytokine bioavailability, based on its capacity to cleave IL‐1β and IL‐6 in vitro [19]. Consequently, loss of *Mmp‐8* enhances antiviral immunity but also amplifies inflammatory responses and necroptosis‐associated tissue injury. Distinct effects on immune regulation may further contribute to these differences. Whereas *Mmp‐9* deficiency was associated with reduced Type I IFN responses and enhanced IFN‐γ‐driven adaptive immunity [19], *Mmp‐8* deficiency resulted in increased Type I IFN levels and augmented PKR/RIP3/MLKL‐mediated necroptosis signaling. Collectively, these findings demonstrate that MMP‐8 and MMP‐9 regulate distinct host defense and immunopathologic pathways during IAV infection.

Current antiviral therapies for IAV primarily target viral proteins such as neuraminidase. In contrast, our data identify MMP‐8 as a host‐derived protease that plays a key role in regulating both viral clearance and immunopathology during IAV infection. Future studies will define the cell‐specific contributions of MMP‐8 and evaluate whether selective modulation of its activity can improve viral control without exacerbating lung injury.

Although broad‐spectrum active site‐directed MMP inhibitors have generally proven unsuitable for the long‐term treatment of chronic diseases because of their limited selectivity among MMP family members, poor oral bioavailability, and dose‐limiting toxicities, these limitations may be less problematic in the setting of acute viral infection that requires only short‐term treatment [47, 48]. Accordingly, the therapeutic potential of transient or cell‐selective MMP‐8 modulation warrants further investigation in severe IAV infection.

Several limitations of this study should be acknowledged. First, the human studies were conducted in relatively small cohorts of subjects with seasonal and H1N1 influenza, which may have limited our ability to account for all potential confounding variables. One limitation of the human cohort is that obesity was more prevalent in influenza‐infected subjects than in healthy controls. Because obesity is a recognized risk factor for severe influenza, it could potentially confound associations between plasma MMP‐8 levels and clinical measures of disease severity. To address this concern, we performed multivariable analyses adjusting for obesity and influenza vaccination status and found that elevated plasma MMP‐8 levels remained significantly associated with lower PaO_2_/FiO_2_ ratios. However, the number of vaccinated subjects was small, limiting statistical power to assess the independent contribution of vaccination status. Taken together, the human data suggest that plasma MMP‐8 may have potential value as a candidate marker of lung injury severity during influenza infection; however, this association requires validation in larger prospective cohorts with balanced enrollment, complete covariate data, standardized sampling times, and longitudinal clinical outcomes.

Second, although *Mmp‐8* deficiency was associated with increased pulmonary Type I IFN protein levels during IAV infection, the mechanism underlying this increase remains unclear and represents a key unresolved question of this study. Type I IFN transcript levels were similar in infected WT and *Mmp‐8* ^*−/−*^ lungs, and MMP‐8 showed only limited cleavage of IFN‐α2 and IFN‐β in vitro. Therefore, the elevated IFN protein levels cannot currently be attributed to increased transcription or loss of direct MMP‐8‐mediated proteolysis. They may instead reflect differences in cellular sources, secretion, extracellular retention, compartmentalization, or the pulmonary microenvironment. These possibilities require direct experimental investigation, and our findings establish an association rather than a direct causal mechanism. In addition, IFN‐β‐induced p‐RIP3 and p‐MLKL signals in primary airway epithelial cells were quantified by immunofluorescence rather than Western blotting; therefore, further validation of these findings will be required in future studies.

Third, although MMP‐8 cleaved IL‐1β and IL‐6 in vitro, the physiological relevance of this activity remains uncertain. The assays used recombinant proteins at relatively high concentrations and an extended incubation time, and we did not measure active MMP‐8 concentrations or detect MMP‐8‐generated cytokine cleavage products in infected lungs. Therefore, these findings raise the possibility that loss of MMP‐8‐mediated cytokine processing contributes to increased pulmonary IL‐1β and IL‐6 levels in *Mmp‐8* ^*−/−*^ mice but do not establish this mechanism in vivo.

Fourth, the use of global *Mmp‐8* knockout mice does not distinguish the relative contributions of MMP‐8 derived from airway epithelial cells, macrophages, and PMNs to viral clearance and lung immunopathology. Therefore, the present findings apply to global *Mmp‐8* deficiency and do not identify the cellular source responsible for the observed phenotype. Future studies using epithelial‐ and myeloid‐specific *Mmp‐8* knockout models will be required to define the cell‐specific functions of MMP‐8 and determine whether selective targeting of particular cellular sources can enhance viral control without exacerbating lung injury.

Finally, the relative contributions of enhanced Type I IFN signaling, increased necroptosis, and augmented adaptive immune responses to the improved viral clearance observed in IAV‐infected *Mmp‐8* ^*−/−*^ mice remain uncertain. Although our data suggest that these pathways may contribute to the observed phenotype, the present study does not establish a complete linear causal pathway from *Mmp‐8* deficiency to increased Type I IFN levels, enhanced necroptosis, and altered viral clearance or lung injury. The extent to which these responses act independently or cooperatively also remains unclear. Future studies employing genetic and pharmacologic approaches will be needed to define the specific mechanisms by which MMP‐8 regulates antiviral immunity and immunopathology during influenza infection. Additionally, the inflammatory mediator analyses involved multiple outcomes and time points without global adjustment across all comparisons, which may increase the risk of Type I error. These findings should therefore be considered exploratory and require independent validation.

Collectively, our findings identify MMP‐8 as a regulator of host responses to IAV infection and a potential host‐response target for severe influenza. *Mmp‐8* deficiency was associated with enhanced Type I IFN‐associated necroptotic signaling and antiviral immune responses, although the upstream mechanism linking MMP‐8 to increased pulmonary Type I IFN protein levels remains unresolved. Therapeutic strategies targeting MMP‐8 will therefore need to balance potential improvements in viral control against the risk of increased immune‐mediated lung injury.

## Author Contributions

X.W. and C.A.O. conceived the project and designed the experiments; X.W., J.R.‐Q., and D.Z. conducted experiments and/or contributed to data analysis. X.W. and C.A.O. contributed to data interpretation. J.Z. collected the samples from human subjects and provided clinical data. X.W. and C.A.O. wrote the manuscript. All authors reviewed and approved the manuscript.

## Funding

This study was supported by HHS | NIH | National Institute of Allergy and Infectious Diseases (NIAID) (AI111475‐01), DOW | MHS | Congressionally Directed Medical Research Programs (CDMRP) (PR152060), Flight Attendant Medical Research Institute (FAMRI) (CIA123046), HHS | NIH | National Institute of Allergy and Infectious Diseases (NIAID) (AI169063), HHS | NIH | National Heart, Lung, and Blood Institute (NHLBI) (F32HL147437), HHS | NIH | National Center for Advancing Translational Sciences (NCATS) (KL2TR002381 and UL1TR002378), and HHS | NIH | National Heart, Lung, and Blood Institute (NHLBI) (HL063137, HL086814, HL111835).

## Conflicts of Interest

During the preparation of this manuscript, Dr. Owen was an employee of Vertex Pharmaceuticals Inc. and subsequently AstraZeneca Biopharmaceuticals R&D. She has no conflicts of interest relevant to the subject of this manuscript. None of the other authors report conflicts of interest.

## Supporting information


**Figure S1:** Interferon‐α, interferon‐β and interferon‐γ steady state mRNA levels are similar in whole lung tissues from infected *Mmp‐8* ^*−/−*^ vs. WT.
**Figure S2:** *Mmp‐8* ^*−/−*^ and WT mice have similar BAL leukocytes, macrophages, PMNs and NK cell counts after H1N1 IAV infection.
**Figure S3:** H1N1 IAV infection induces similar increases in lung levels of Ccl‐3, Cxcl‐2, Gm‐csf, M‐csf, Il‐17, Tnf‐α, Il‐10, Tgf‐β in *Mmp‐8* ^*−/−*^ and WT mice.
**Table S1:** Sequences of the primers used for real‐time PCR.

## Data Availability

All data supporting the findings of this study are included in the article and its Supporting Information.

## References

[1] F. Krammer , G. J. D. Smith , R. A. M. Fouchier , et al., “Influenza,” Nature Reviews Disease Primers 4 (2018): 3. 10.1038/s41572-018-0002-y PMC7097467 29955068

[2] M. Crespo , S. Collado , M. Mir , et al., “Efficacy of Influenza A H1N1/2009 Vaccine in Hemodialysis and Kidney Transplant Patients,” Clinical Journal of the American Society of Nephrology 6 (2011): 2208–2214. 10.2215/CJN.02160311 PMC3358996 21852661

[3] J. A. Englund , “Antiviral Therapy of Influenza,” Seminars in Pediatric Infectious Diseases 13 (2002): 120–128. 10.1053/spid.2002.122999 12122950

[4] M. Crusat and M. D. de Jong , “Neuraminidase Inhibitors and Their Role in Avian and Pandemic Influenza,” Antiviral Therapy 12 (2007): 593–602. 17944267

[5] C. A. Owen , Z. Hu , C. Lopez‐Otin , and S. D. Shapiro , “Membrane‐Bound Matrix Metalloproteinase‐8 on Activated Polymorphonuclear Cells Is a Potent, Tissue Inhibitor of Metalloproteinase‐Resistant Collagenase and Serpinase,” Journal of Immunology 172 (2004): 7791–7803. 10.4049/jimmunol.172.12.7791 15187163

[6] P. A. Quintero , M. D. Knolle , L. F. Cala , Y. Zhuang , and C. A. Owen , “Matrix Metalloproteinase‐8 Inactivates Macrophage Inflammatory Protein‐1 Alpha to Reduce Acute Lung Inflammation and Injury in Mice,” Journal of Immunology 184 (2010): 1575–1588. 10.4049/jimmunol.0900290 PMC2938777 20042585

[7] V. J. Craig , P. A. Quintero , S. E. Fyfe , et al., “Profibrotic Activities for Matrix Metalloproteinase‐8 During Bleomycin‐Mediated Lung Injury,” Journal of Immunology 190 (2013): 4283–4296. 10.4049/jimmunol.1201043 PMC3639121 23487425

[8] X. Wang , J. Rojas‐Quintero , J. Wilder , Y. Tesfaigzi , D. Zhang , and C. A. Owen , “Tissue Inhibitor of Metalloproteinase‐1 Promotes Polymorphonuclear Neutrophil (PMN) Pericellular Proteolysis by Anchoring Matrix Metalloproteinase‐8 and ‐9 to PMN Surfaces,” Journal of Immunology 202 (2019): 3267–3281. 10.4049/jimmunol.1801466 PMC7347292 31019060

[9] V. J. Craig , F. Polverino , M. E. Laucho‐Contreras , et al., “Mononuclear Phagocytes and Airway Epithelial Cells: Novel Sources of Matrix Metalloproteinase‐8 (MMP‐8) in Patients With Idiopathic Pulmonary Fibrosis,” PLoS One 9 (2014): e97485. 10.1371/journal.pone.0097485 PMC4020836 24828408

[10] M. P. Herman , G. K. Sukhova , P. Libby , et al., “Expression of Neutrophil Collagenase (Matrix Metalloproteinase‐8) in Human Atheroma: A Novel Collagenolytic Pathway Suggested by Transcriptional Profiling,” Circulation 104 (2001): 1899–1904. 10.1161/hc4101.097419 11602491

[11] X. Wang , D. Zhang , Q. A. Fucci , C. M. Dollery , and C. A. Owen , “Surface‐Bound Matrix Metalloproteinase‐8 on Macrophages: Contributions to Macrophage Pericellular Proteolysis and Migration Through Tissue Barriers,” Physiological Reports 9 (2021): e14778. 10.14814/phy2.14778 PMC7927794 33656791

[12] R. E. Vandenbroucke , E. Dejonckheere , P. Van Lint , et al., “Matrix Metalloprotease 8‐Dependent Extracellular Matrix Cleavage at the Blood‐CSF Barrier Contributes to Lethality During Systemic Inflammatory Diseases,” Journal of Neuroscience 32 (2012): 9805–9816. 10.1523/JNEUROSCI.0967-12.2012 PMC6621276 22815495

[13] X. Zhou , J. Lu , D. Chen , et al., “Matrix Metalloproteinase‐8 Inhibitors Mitigate Sepsis‐Induced Myocardial Injury in Rats,” Chinese Medical Journal (English) 127 (2014): 1530–1535. 24762601

[14] A. R. Folgueras , A. Fueyo , O. Garcia‐Suarez , et al., “Collagenase‐2 Deficiency or Inhibition Impairs Experimental Autoimmune Encephalomyelitis in Mice,” Journal of Biological Chemistry 283 (2008): 9465–9474. 10.1074/jbc.M709522200 18245084

[15] J. H. Cox , A. E. Starr , R. Kappelhoff , R. Yan , C. R. Roberts , and C. M. Overall , “Matrix Metalloproteinase 8 Deficiency in Mice Exacerbates Inflammatory Arthritis Through Delayed Neutrophil Apoptosis and Reduced Caspase 11 Expression,” Arthritis and Rheumatism 62 (2010): 3645–3655. 10.1002/art.27757 21120997

[16] M. Balbin , A. Fueyo , A. M. Tester , et al., “Loss of Collagenase‐2 Confers Increased Skin Tumor Susceptibility to Male Mice,” Nature Genetics 35 (2003): 252–257. 10.1038/ng1249 14517555

[17] M. M. Gueders , M. Balbin , N. Rocks , et al., “Matrix Metalloproteinase‐8 Deficiency Promotes Granulocytic Allergen‐Induced Airway Inflammation,” Journal of Immunology 175 (2005): 2589–2597. 10.4049/jimmunol.175.4.2589 16081833

[18] D. Talmi‐Frank , Z. Altboum , I. Solomonov , et al., “Extracellular Matrix Proteolysis by MT1‐MMP Contributes to Influenza‐Related Tissue Damage and Mortality,” Cell Host & Microbe 20 (2016): 458–470. 10.1016/j.chom.2016.09.005 27736644

[19] J. Rojas‐Quintero , X. Wang , J. Tipper , et al., “Matrix Metalloproteinase‐9 Deficiency Protects Mice From Severe Influenza A Viral Infection,” JCI Insight 3 (2018): e99022. 10.1172/jci.insight.99022 PMC6338318 30568032

[20] J. Zuniga , I. Buendia‐Roldan , Y. Zhao , et al., “Genetic Variants Associated With Severe Pneumonia in A/H1N1 Influenza Infection,” European Respiratory Journal 39 (2012): 604–610. 10.1183/09031936.00020611 PMC3816497 21737555

[21] M. Brandes , F. Klauschen , S. Kuchen , and R. N. Germain , “A Systems Analysis Identifies a Feedforward Inflammatory Circuit Leading to Lethal Influenza Infection,” Cell 154 (2013): 197–212. 10.1016/j.cell.2013.06.013 PMC3763506 23827683

[22] X. Wang , F. Polverino , J. Rojas‐Quintero , et al., “A Disintegrin and A Metalloproteinase‐9 (ADAM9): A Novel Proteinase Culprit With Multifarious Contributions to COPD,” American Journal of Respiratory and Critical Care Medicine 198, no. 12 (2018): 1500–1518. 10.1164/rccm.201711-2300OC PMC6298633 29864380

[23] O. Dienz , J. G. Rud , S. M. Eaton , et al., “Essential Role of IL‐6 in Protection Against H1N1 Influenza Virus by Promoting Neutrophil Survival in the Lung,” Mucosal Immunology 5 (2012): 258–266. 10.1038/mi.2012.2 PMC3328598 22294047

[24] M. E. Laucho‐Contreras , K. L. Taylor , R. Mahadeva , S. S. Boukedes , and C. A. Owen , “Automated Measurement of Pulmonary Emphysema and Small Airway Remodeling in Cigarette Smoke‐Exposed Mice,” Journal of Visualized Experiments (2015): 52236, 10.3791/52236. PMC4354540 25651034

[25] X. A. Han , H. Y. Jie , J. H. Wang , et al., “Necrostatin‐1 Ameliorates Neutrophilic Inflammation in Asthma by Suppressing MLKL Phosphorylation to Inhibiting NETs Release,” Frontiers in Immunology 11 (2020): 666. 10.3389/fimmu.2020.00666 PMC7194114 32391007

[26] B. Lin , Z. Jin , X. Chen , et al., “Necrostatin‐1 Protects Mice From Acute Lung Injury by Suppressing Necroptosis and Reactive Oxygen Species,” Molecular Medicine Reports 21 (2020): 2171–2181. 10.3892/mmr.2020.11010 PMC7115190 32323764

[27] E. J. Campbell and C. A. Owen , “The Sulfate Groups of Chondroitin Sulfate‐ and Heparan Sulfate‐Containing Proteoglycans in Neutrophil Plasma Membranes Are Novel Binding Sites for Human Leukocyte Elastase and Cathepsin G,” Journal of Biological Chemistry 282 (2007): 14645–14654. 10.1074/jbc.M608346200 17384412

[28] H. C. Lam , A. M. Choi , and S. W. Ryter , “Isolation of Mouse Respiratory Epithelial Cells and Exposure to Experimental Cigarette Smoke at Air Liquid Interface,” Journal of Visualized Experiments (2011), 10.3791/2513. PMC3197407 21372793

[29] R. J. Thapa , S. Nogusa , P. Chen , et al., “Interferon‐Induced RIP1/RIP3‐Mediated Necrosis Requires PKR and Is Licensed by FADD and Caspases,” Proceedings of the National Academy of Sciences of the United States of America 110 (2013): E3109–E3118. 10.1073/pnas.1301218110 PMC3746924 23898178

[30] K. Mizumura , S. M. Cloonan , K. Nakahira , et al., “Mitophagy‐Dependent Necroptosis Contributes to the Pathogenesis of COPD,” Journal of Clinical Investigation 124 (2014): 3987–4003. 10.1172/JCI74985 PMC4151233 25083992

[31] L. Galluzzi , I. Vitale , S. A. Aaronson , et al., “Molecular Mechanisms of Cell Death: Recommendations of the Nomenclature Committee on Cell Death 2018,” Cell Death and Differentiation 25 (2018): 486–541. 10.1038/s41418-017-0012-4 PMC5864239 29362479

[32] H. Zhao , T. Jaffer , S. Eguchi , Z. Wang , A. Linkermann , and D. Ma , “Role of Necroptosis in the Pathogenesis of Solid Organ Injury,” Cell Death & Disease 6 (2015): e1975. 10.1038/cddis.2015.316 PMC4670925 26583318

[33] C. E. Samuel , “Antiviral Actions of Interferons,” Clinical Microbiology Reviews 14 (2001): 778–809, table of contents. 10.1128/CMR.14.4.778-809.2001 PMC89003 11585785

[34] A. Iwasaki and P. S. Pillai , “Innate Immunity to Influenza Virus Infection,” Nature Reviews. Immunology 14 (2014): 315–328. 10.1038/nri3665 PMC4104278 24762827

[35] O. Haller , G. Kochs , and F. Weber , “The Interferon Response Circuit: Induction and Suppression by Pathogenic Viruses,” Virology 344 (2006): 119–130. 10.1016/j.virol.2005.09.024 PMC7125643 16364743

[36] S. Ghosh , D. Gregory , A. Smith , and L. Kobzik , “MARCO Regulates Early Inflammatory Responses Against Influenza: A Useful Macrophage Function With Adverse Outcome,” American Journal of Respiratory Cell and Molecular Biology 45 (2011): 1036–1044. 10.1165/rcmb.2010-0349OC PMC3262690 21562316

[37] I. Nelissen , E. Martens , P. E. Van den Steen , P. Proost , I. Ronsse , and G. Opdenakker , “Gelatinase B/Matrix Metalloproteinase‐9 Cleaves Interferon‐Beta and Is a Target for Immunotherapy,” Brain 126 (2003): 1371–1381. 10.1093/brain/awg129 12764058

[38] T. Peiro , D. F. Patel , S. Akthar , et al., “Neutrophils Drive Alveolar Macrophage IL‐1beta Release During Respiratory Viral Infection,” Thorax 73 (2018): 546–556. 10.1136/thoraxjnl-2017-210010 PMC5969338 29079611

[39] N. Schmitz , M. Kurrer , M. F. Bachmann , and M. Kopf , “Interleukin‐1 Is Responsible for Acute Lung Immunopathology but Increases Survival of Respiratory Influenza Virus Infection,” Journal of Virology 79 (2005): 6441–6448. 10.1128/JVI.79.10.6441-6448.2005 PMC1091664 15858027

[40] S. N. Lauder , E. Jones , K. Smart , et al., “Interleukin‐6 Limits Influenza‐Induced Inflammation and Protects Against Fatal Lung Pathology,” European Journal of Immunology 43 (2013): 2613–2625. 10.1002/eji.201243018 PMC3886386 23857287

[41] H. S. Lee , J. Y. Noh , O. S. Shin , J. Y. Song , H. J. Cheong , and W. J. Kim , “Matrix Metalloproteinase‐13 in Atherosclerotic Plaque Is Increased by Influenza A Virus Infection,” Journal of Infectious Diseases 221 (2020): 256–266. 10.1093/infdis/jiz580 31693113

[42] B. Villeret , B. Solhonne , M. Straube , et al., “Influenza A Virus Pre‐Infection Exacerbates *Pseudomonas aeruginosa* ‐Mediated Lung Damage Through Increased MMP‐9 Expression, Decreased Elafin Production and Tissue Resilience,” Frontiers in Immunology 11 (2020): 117. 10.3389/fimmu.2020.00117 PMC7031978 32117268

[43] B. M. Tang , M. Shojaei , S. Teoh , et al., “Neutrophils‐Related Host Factors Associated With Severe Disease and Fatality in Patients With Influenza Infection,” Nature Communications 10 (2019): 3422. 10.1038/s41467-019-11249-y PMC6668409 31366921

[44] L. J. Rosenwasser , “Biologic Activities of IL‐1 and Its Role in Human Disease,” Journal of Allergy and Clinical Immunology 102 (1998): 344–350. 10.1016/s0091-6749(98)70118-6 9768571

[45] A. C. Bolognese , W. L. Yang , L. W. Hansen , et al., “Inhibition of Necroptosis Attenuates Lung Injury and Improves Survival in Neonatal Sepsis,” Surgery 164 (2018): 110–116. 10.1016/j.surg.2018.02.017 PMC6204110 29709367

[46] J. R. Teijaro , K. B. Walsh , S. Rice , H. Rosen , and M. B. Oldstone , “Mapping the Innate Signaling Cascade Essential for Cytokine Storm During Influenza Virus Infection,” Proceedings of the National Academy of Sciences of the United States of America 111 (2014): 3799–3804. 10.1073/pnas.1400593111 PMC3956176 24572573

[47] M. Selman , J. Cisneros‐Lira , M. Gaxiola , et al., “Matrix Metalloproteinases Inhibition Attenuates Tobacco Smoke‐Induced Emphysema in Guinea Pigs,” Chest 123 (2003): 1633–1641. 10.1378/chest.123.5.1633 12740284

[48] K. Kaneko , R. O. Williams , D. T. Dransfield , A. E. Nixon , A. Sandison , and Y. Itoh , “Selective Inhibition of Membrane Type 1 Matrix Metalloproteinase Abrogates Progression of Experimental Inflammatory Arthritis: Synergy With Tumor Necrosis Factor Blockade,” Arthritis & Rhematology 68 (2016): 521–531. 10.1002/art.39414 PMC4738413 26315469

