# Supplementary material for: Matrix Metalloproteinase‐8 Deficiency Alters the Balance Between Viral Clearance and Immunopathology in H1N1 Infection
Source: FASEB J. 2026 Oct 12;40(19):e72372. doi: 10.1096/fj.202601719RRR (PMC13656188; doi:10.1096/fj.202601719RRR)
Supplement: Supplementary file 1 [file FSB2-40-e72372-s001.docx]

**ONLINE SUPPLEMENT**

**Figure S1: Interferon-α, interferon-β and interferon-γ steady state mRNA levels are similar in whole lung tissues from infected *Mmp-8^-/-^* vs. WT.** Interferon-α2 (**A**), interferon-α4 (**B**), interferon-β (**C**) and interferon-γ (**D**) steady state mRNA levels were measured in lungs obtained from uninfected WT and *Mmp-8^-/-^* (5-7 mice per group), and WT and *Mmp-8^-/-^* infected with 10^3^ PFU H1N1 IAV at intervals up to 10 days post infection using RT-PCR (6-8 mice per group). There were no significant differences in interferon-α, interferon-β and interferon-γ transcriptional levels in WT or *Mmp-8^-/-^* at any time-point post infection.

**Figure S2: *Mmp-8^-/-^* and WT mice have similar BAL leukocytes, macrophages, PMNs and NK cell counts after H1N1 IAV infection.** WT and *Mmp-8^-/-^* mice (n = 4-10 mice per group) were infected with 10^3^ PFU H1N1 IAV *via* the intranasal route, or not infected as a control. BAL was performed on the mice at intervals p.i. Leukocytes (**A**), macrophages (**B**), PMNs (**C**) were counted in BAL samples. BAL leukocytes were immunostained for markers of PMNs (**D**), macrophages (**E**), and NK cells (**F**), and analyzed with flow cytometry. The boxes in the box-plots show the medians and 25^th^ and 75^th^ percentiles, and the whiskers show the 10^th^ and 90^th^ percentiles. Data were analyzed using a Kruskal-Wallis One-Way ANOVA test followed by pair-wise testing with Mann-Whitney U tests.

**Figure S3: H1N1 IAV infection induces similar increases in lung levels of Ccl-3, Cxcl-2, Gm-csf, M-csf, Il-17, Tnf-α, Il-10, Tgf-β in *Mmp-8^-/-^* and WT mice.** WT and *Mmp-8^-/-^* mice were infected with 10^3^ PFU H1N1 *via* the i.n. route. At intervals p.i., lung levels of Ccl-3 (**A**, n = 4-10 mice per group), Cxcl-2 (**B**, n = 4-9 mice per group), Gm-csf (**C**, n = 6-8 mice per group), M-csf (**D**, n = 6-8 mice per group), Il-17 (**E**, n = 6-8 mice per group), Tnf-α (**F**, n = 4-10 mice per group), Il-10 (**G**, n = 4-10 mice per group) and Tgf-β (**H**, n = 4-8 mice per group) were measured using ELISAs and normalized to lung total protein levels. Boxes in the box-plots show the medians and 25^th^ and 75^th^ percentiles, and the whiskers show the 10^th^ and 90^th^ percentiles. Data were analyzed using a Kruskal-Wallis One-Way ANOVA followed by pair-wise testing with Mann-Whitney U tests.

**Table S1: Sequences of the primers used for Real-time PCR.**

| **Gene** | **Forward primer** | **Reverse primer** |
| --- | --- | --- |
| ***Ppia*** | GAGCTGTTTGCAGACAAAGTTC | CCCTGGCACATGAATCCTGG |
| ***β-Actin*** | AGTGTGACGTTGACATCCGT | TGCTAGGAGCCAGAGCAGTA |
| ***Mmp-8*** | AATCCTTGCCCATGCCTTTCAACC | CCAAATTCATGAGCAGCCACGAGA |
| ***H1N1*** | TTCACAGCATCGGTCTCACAGACA | TCCAGCCATCTGTTCCATAGCCTT |
| ***Interferon-α2*** | TTGAAGGTCCTGGCACAG | GAGGTTCAAGGTCTGCTGA |
| ***Interferon-α4*** | CCTGTGTGATGCAGGAACC | TCACCTCCCAGGCACAGA |
| ***Interferon-β*** | CAGCTCCAAGAAAGGACGAAC | GGCAGTGTAACTCTTCTGCAT |
| ***Interferon-γ*** | ATGAACGCTACACACTGCATC | CCATCCTTTTGCCAGTTCCTC |
| ***Eif2ak2*** | ATGCACGGAGTAGCCATTACG | TGACAATCCACCTTGTTTTCGT |
| ***Rip1*** | GAAGACAGACCTAGACAGCGG | CCAGTAGCTTCACCACTCGAC |
| ***Rip3*** | TCTGTCAAGTTATGGCCTACTGG | GGAACACGACTCCGAACCC |

The Table shows the sequences of the primers that were used to perform quantitative real-time RT-PCR to measure Mmp-8, interferon-α, interferon-β, interferon-γ, Eif2ak2 (Pkr), Rip1 and Rip3 gene expression and viral titers in murine lung samples from uninfected mice and H1N1 IAV-infected mice.
